# Teaching diffusion models physics: reinforcement learning for physically valid diffusion-based docking

**DOI:** 10.1039/d6sc02655a

**Published:** 2026-08-18

**Authors:** J. Henry Broster, Bojana Popovic, Diana Kondinskaia, Charlotte M. Deane, Fergus Imrie

**Affiliations:** a Department of Statistics, University of Oxford Oxford OX1 3LB UK imrie@stats.ox.ac.uk; b Cambridge Crystallographic Data Centre Cambridge CB2 1EZ UK

## Abstract

Molecular docking aims to predict the binding conformation of a small molecule to its protein target. Recent work has proposed diffusion models for this task, from rigid-body docking that diffuses over ligand degrees of freedom to co-folding approaches that jointly generate protein structure and ligand pose. However, diffusion-based docking models have been shown to frequently produce physically implausible poses and fail to consistently recover key protein-ligand interactions. To address this, we introduce a reinforcement learning framework for training diffusion-based docking models directly on non-differentiable objectives. Fine-tuning DiffDock-Pocket for physical validity with our approach substantially increases the number of generated poses that are physically valid and interaction-preserving, with no increase in inference-time compute. Importantly, this comes without sacrificing structural accuracy; in fact, our approach increases the proportion of structures with near-native poses. These effects are most pronounced for protein targets that are dissimilar to the training data. Our fine-tuned DiffDock-Pocket model outperforms both classical docking algorithms and machine learning-based approaches on the PoseBusters set. Our results demonstrate that reinforcement learning can teach diffusion-based docking models to better respect physical constraints and recover key interactions, without the requirement to rely on inference-time corrections.

## Abstract 2

Reinforcement learning can be used to teach diffusion models to generate physically valid, interaction-preserving poses without increasing inference compute.

## Graphical abstract

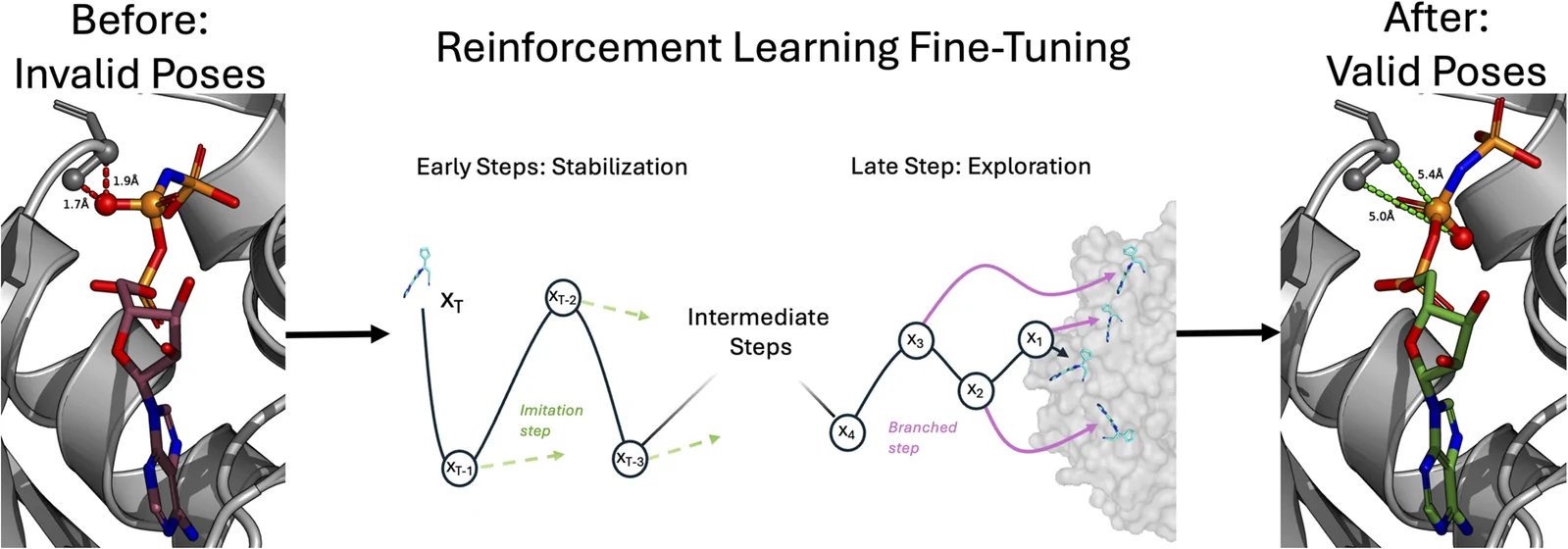

## 1 Introduction

Drug discovery remains slow, costly, and marked by high late-stage attrition.^1,2^ This motivates computational methods that can more reliably prioritize candidates before expensive experimental and clinical testing.^1^ Structure-based drug design (SBDD) exploits three-dimensional (3D) information about the target and ligand structures to identify, design, and optimize candidate molecules.^3^ A key component of SBDD is molecular docking, which predicts the ligand orientation and conformation in the binding site and is widely used for virtual screening and lead optimization.^4,5^ Docking can reduce experimental burden by prioritizing compounds more likely to succeed, but in practice prospective hit rates are often low and can vary substantially across targets and screening setups.^6–9^

Classical approaches to molecular docking rely on physics-inspired scoring functions combined with heuristic or stochastic search algorithms. Docking tools such as AutoDock Vina,^10^ Glide,^11^ and GOLD^12^ generate poses by searching over the translational, torsional, and rotational space that the ligand can take using, for instance, a genetic algorithm before ranking them using physics-based scoring functions, which serve as a proxy for binding free energy. More recently, machine learning models have increasingly been adopted to replace either the search or scoring components of docking. Early machine learning (ML)-based approaches, such as GNINA,^13^ used 3D convolutional neural networks to rescore poses generated with classical algorithms, while regression-based methods such as EquiBind,^14^ TankBind,^15^ CarsiDock,^16^ and ArtiDock^17^ operate directly in 3D space to predict ligand poses. These models are trained with supervised regression objectives, producing pose predictions that may average over multiple valid binding modes rather than capturing any one accurately. As a result, they do not model the inherent multimodality of protein-ligand binding, motivating the use of diffusion-based generative models such as DiffDock,^18^ DiffDock-Pocket,^19^ DiffBindFR,^20^ and SurfDock,^21^ which have been shown to better model multimodal distributions.^18,22^

Diffusion generative models^23,24^ are a class of machine learning models that learn complex data distributions by reversing a stochastic noise process. Diffusion models define a forward process that gradually corrupts data with noise and learn a reverse process that iteratively removes this noise, generating new samples from the learned distribution. In the context of molecular docking, this framework can be used to model distributions over protein–ligand poses. When initialized from random noise, the reverse diffusion process produces plausible binding configurations within a protein binding pocket.^18,25,26^ Some notable examples of diffusion-based models for structure-based drug design include DiffDock^18,25^ and DiffDock-Pocket^19^ for docking, DiffSBDD^27^ and MolSnapper^28^ for structure-based ligand generation, and Boltz^29^ and AlphaFold3^26^ for co-folding and protein structure prediction. Root-mean-square deviation (RMSD) measures the average distance between corresponding atoms of two molecular structures and is commonly used as a metric for evaluating docking accuracy.

Although diffusion-based docking models have reported strong docking performance under this metric, studies have shown that these machine-learning-based docking methods frequently generate physically implausible structures, even when the predicted poses satisfy the conventional RMSD ≤ 2 Å threshold relative to the experimentally determined binding pose.^30,31^ For example, on the 428-complex PoseBusters Benchmark set, CarsiDock decreases from 79.7% to 47.7% when predictions are also required to be PoseBusters-valid, while SurfDock drops from just below 80% to around 40% under the same validity requirement.^16,21^ On the 344-complex PLINDER test set, ArtiDock reports that 65% of its top-ranked predictions were PoseBusters-valid.^17^ Finally, on the original 308-complex PoseBusters benchmark, DiffDock decreases from 38.0% for RMSD ≤ 2 Å to 12.3% when PoseBusters validity is also required.^30^

Similarly, diffusion-based docking models fail to consistently recover key protein–ligand interactions that can be essential for downstream lead optimization and assessing the quality of hits.^32,33^ These flaws indicate that models have not truly understood the mechanics of binding and highlight the need for approaches that explicitly incorporate criteria such as physical validity and interaction recovery in the training procedure.

We hypothesize that these issues stem from a misalignment between the standard diffusion training objective, which minimizes mean squared error on the added noise (score matching), and the downstream criteria that matter in practice, which include not only geometric accuracy as measured by RMSD but also physical plausibility and recovery of functionally important interactions. The diffusion objective optimizes physical validity and interaction recovery only indirectly, *via* their correlation with the training data distribution, and provides no explicit incentive to avoid physically implausible regions or preserve key interactions. For molecular docking, minimizing RMSD is not strictly equivalent to improving pose quality: while an RMSD of 0 Å would reproduce experimentally determined interactions and be physically valid (given a correct reference), reducing RMSD alone does not guarantee better interaction recovery or physical plausibility (Fig. 1). For example, poses below 2 Å RMSD can still exhibit severe steric clashes or recover few interactions, indicating that geometric proximity does not uniquely determine physical validity or functional correctness.

**Fig. 1:**
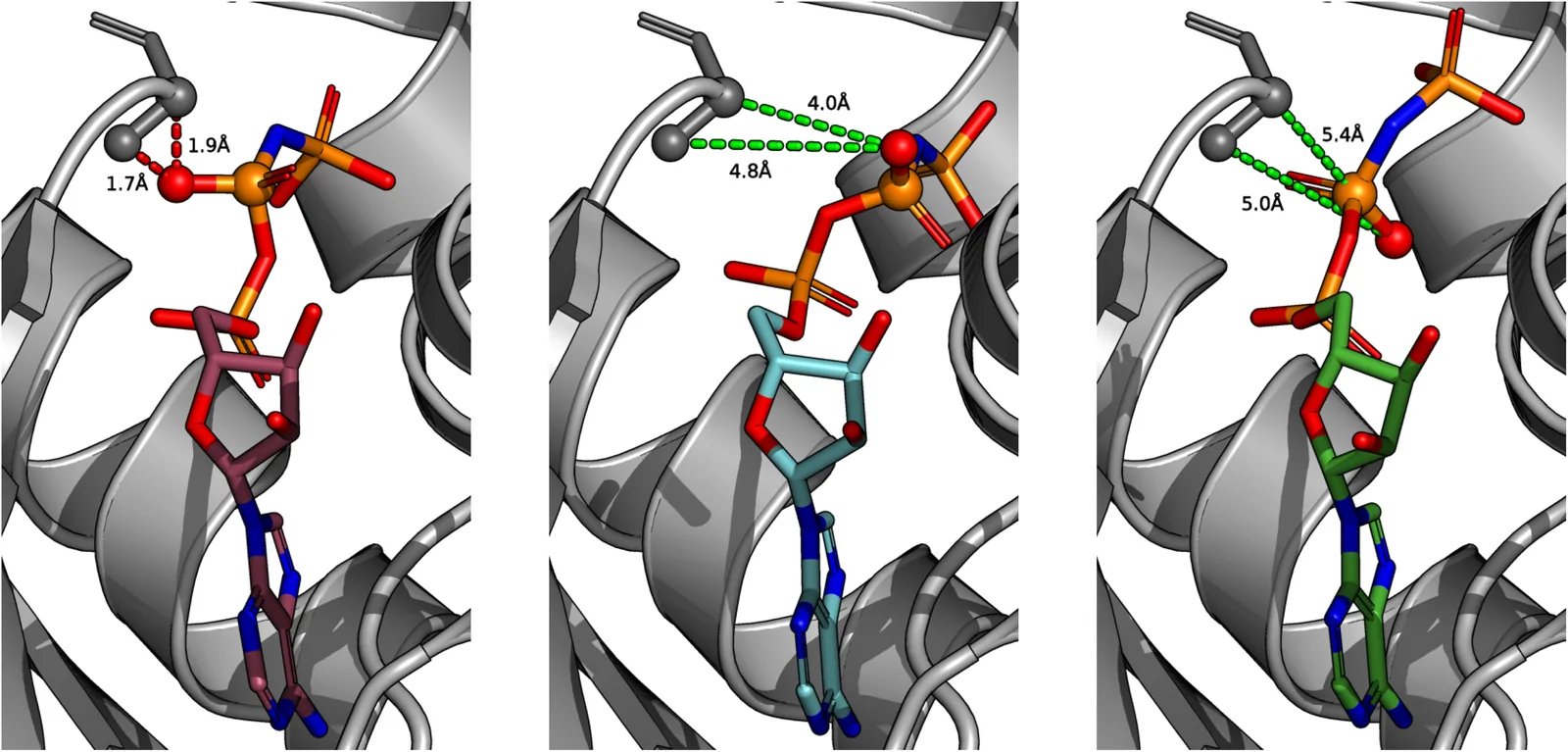
RMSD alone does not guarantee physical plausibility. Three poses are shown for a protein–ligand complex from the PoseBusters benchmark: a 1.4 Å RMSD prediction with a steric clash, the experimentally determined structure (PDB ID: 7B94, ligand ANP), and a 1.7 Å RMSD prediction without a steric clash. Despite the clashing prediction having the lower RMSD, it forms severe protein–ligand steric clashes with the GLY77 backbone, whereas the higher-RMSD prediction avoids these clashes. This example illustrates that minimizing RMSD alone does not guarantee physically plausible binding geometries.

To address this gap, we introduce a reinforcement learning (RL) framework for fine-tuning diffusion-based docking models on non-differentiable downstream objectives, such as physical validity and interaction recovery. Our approach builds on recent work casting diffusion denoising as a Markov decision process that can be optimized with policy-gradient methods,^34^ and introduces two key innovations: early-step imitation regularization, which stabilizes training by biasing initial denoising steps toward ground-truth poses, and late-step trajectory branching, which amplifies learning signals through additional evaluations of the local refinements that often determine pose quality. We demonstrate our approach using a reward function that favors all physically plausible, interaction-preserving conformations within 2 Å RMSD of the ground truth.

Applying our framework to DiffDock-Pocket^19^ (yielding “DiffDock-Pocket RL”) substantially increases the fraction of near-native poses that are physically valid. Since DiffDock-Pocket RL uses the same sampling procedure as the original DiffDock-Pocket model and differs only in the learned score-model weights, this improvement comes at no additional inference-time computational cost. Across all complexes, PoseBusters validity (PB-validity) increases from 58.8% to 78.1% for the top-ranked pose, and from 38.2% to 58.9% across all sampled poses. This effect is most pronounced at low sequence identity (0–30%), where the proportion of PB-valid samples increases from 24.3% to 46.4%. Despite not explicitly optimizing for this objective, the average Vina energy across generated poses improves from 2.24 kcal mol^−1^ to −2.10 kcal mol^−1^, consistent with the model sampling more physically and energetically favorable conformations. Under stricter joint criteria, Oracle success for poses that are both ≤2 Å RMSD to the ground truth and PB-Valid increases from 66.1% to 79.9%. DiffDock-Pocket RL outperforms the original DiffDock-Pocket and established physics-based methods on the PoseBusters benchmark set when evaluation requires physically plausible near-native poses. RL increases Top-1 success for the joint criterion ≤2 Å RMSD & PB-valid, and yields the strongest performance when additionally requiring ≥50% interaction recovery. Improvements come not only from generating more physically valid poses, but also from sampling near-native poses that the baseline fails to reach. This shows that reinforcement learning increases both physical validity and the likelihood of producing accurate, near-native binding modes. When combined with physics-based local minimization and re-ranking, DiffDock-Pocket RL achieves 80.2% of Top-1 predictions with RMSD ≤ 2 Å and 78.2% when physical validity is additionally required, substantially surpassing both physics-based and ML-based docking approaches on the PoseBusters set.

## 2 Methods

We propose a reinforcement learning framework for fine-tuning diffusion-based docking models using non-differentiable objectives, specifically to reward physically valid poses. Our approach builds on Deep Denoising Policy Optimization (DDPO),^34^ which casts the reverse diffusion process as a Markov decision process. In this formulation, each denoising step is treated as an action, and the policy is optimized with respect to a reward defined on the final output using policy-gradient methods. This reframing allows the model to be trained directly on task-relevant criteria, such as physical validity, that are difficult to express through standard likelihood-based objectives. We introduce two key innovations over the standard DDPO framework: (1) early-step imitation regularization to stabilize training, and (2) late-step trajectory branching to provide a more informative learning signal.

### 2.1 Diffusion-based docking with DiffDock-Pocket

We use the publicly available rigid-receptor implementation of DiffDock-Pocket, in which the receptor pocket is fixed and only the ligand translation, rotation, and torsion degrees of freedom are updated during denoising. DiffDock-Pocket defines a diffusion model over the ligand pose degrees of freedom 
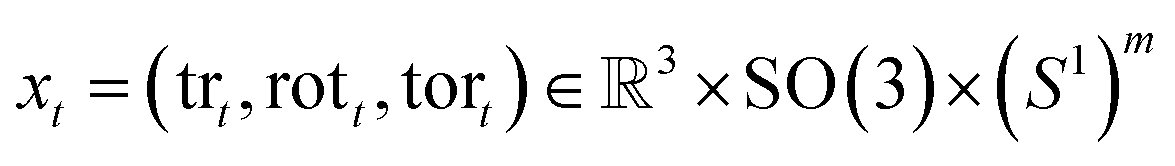
, where 
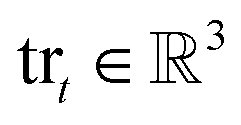
 is the ligand translation, rot_*t*_ ∈ SO(3) the rotation, and tor_*t*_ ∈ (*S*^1^)^*m*^ the *m* torsion angles, conditioned on the protein pocket representation *c*. In the forward process, each component is perturbed by independent Brownian motion with diffusion schedule *g*_*κ*_(*t*), for *κ* ∈ {tr, rot, tor}. The learned reverse process is specified by score functions *f*_*κ*_(*x*_*t*_, *c*; *θ*) in the corresponding tangent spaces.

In discrete time, the reverse transition is written in Euler–Maruyama form as Gaussian updates in the tangent coordinates for each component, where Δ*t* denotes the timestep size:
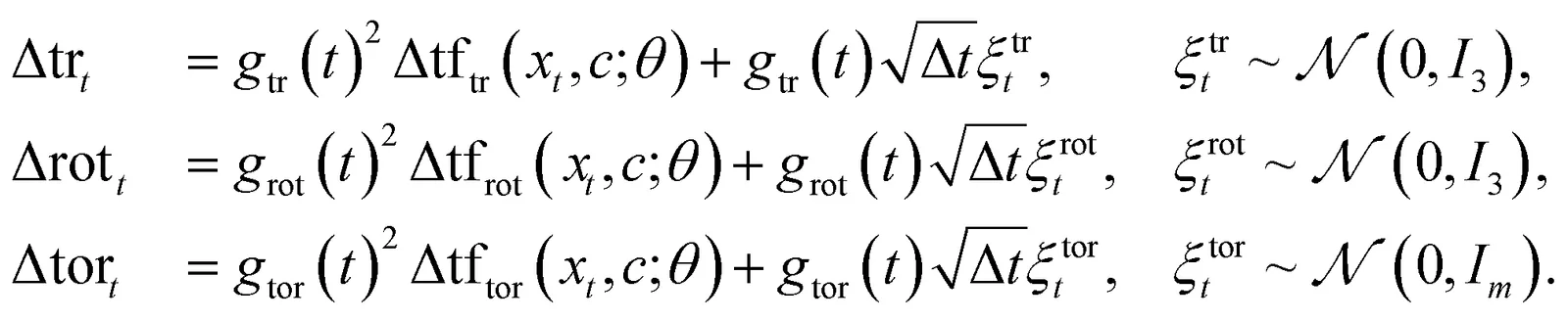


These increments are applied to update the pose as
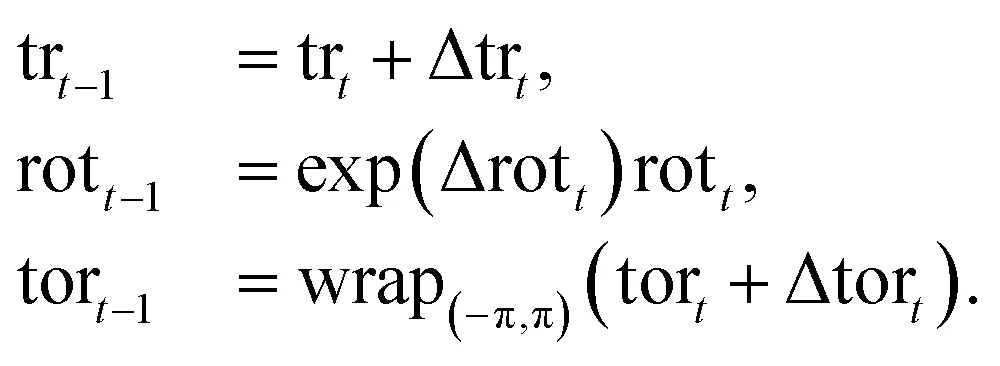


Together, these updates define a Markov transition *p*_*θ*_(*x*_*t*−1_|*x*_*t*_, *c*), with mean drift determined by the learned scores *f*_*κ*_ and stochasticity from the Gaussian noises ξ^*κ*^_*t*_. Since the reverse transition defines a stochastic distribution over pose updates, it can be interpreted as a policy. This makes it possible to optimize parameters *θ* with reinforcement learning to increase the probability of trajectories that terminate in high-reward, physically valid poses.

### 2.2 Reverse diffusion as a Markov decision process

We formulate the reverse diffusion process as a Markov decision process (MDP), {*S*, *A*, *P*, *R*, *γ*}, over timesteps *t* = *T*, *T* − 1, …, 0 as follows.

#### 2.2.1 State space (*S*)

The state at time *t* is given by *s*_*t*_ = (*x*_*t*_, *c*), where *x*_*t*_ = (tr_*t*_, rot_*t*_, tor_*t*_) represents the ligand pose at timestep *t* with translation 
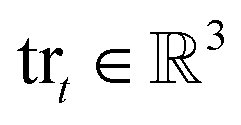
, rotation rot_*t*_ ∈ SO(3), and torsion angles tor_*t*_ ∈ (*S*^1^)^*m*^, and *c* is the conditioning protein structure.

#### 2.2.2 Action space (*A*)

The action at time *t* is given by *a*_*t*_ = (Δtr_*t*_, Δrot_*t*_, Δtor_*t*_), where 
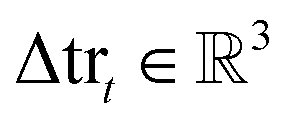
 is the translational update, 
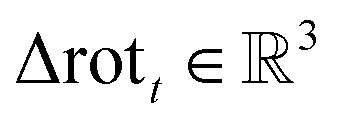
 is an axis-angle rotation update applied *via* rot_*t*−1_ = exp(Δrot_*t*_)rot_*t*_, and 
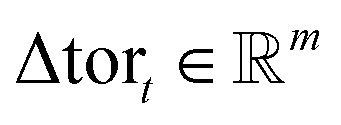
 are the *m* torsion updates, with torsion angles wrapped to (−π, π) after application.

#### 2.2.3 Transition dynamics (*P*)

Given state *s*_*t*_ and (sampled) action *a*_*t*_, the next state *s*_*t*−1_ is obtained by applying the ligand pose updates from Section 2.1 deterministically, *i.e. P*(*s*_*t*−1_|*s*_*t*_,*a*_*t*_) = *δ*_*u*(*s*_*t*_,*a*_*t*_)_, where *u* denotes the deterministic pose update and *δ* represents the Dirac delta function. All stochasticity in the MDP therefore arises from the policy, not the environment.

#### 2.2.4 Reward (*R*)

The reward *R* is based on the final state *s*_0_ = (*x*_0_, *c*) and zero otherwise, *i.e.*
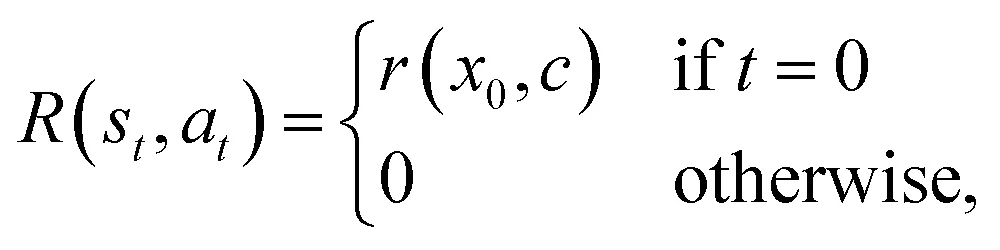
and thus the cumulative reward of each trajectory is equal to *r*(*x*_0_, *c*). Full details of the reward function are given in Section 2.6.

#### 2.2.5 Discount factor (*γ*)

Since rewards are zero except at *t* = 0, the discounted return at timestep *t* in a trajectory is *γ*^*t*^*r*(*x*_0_, *c*) for *γ* ∈ [0, 1].

#### 2.2.6 Policy (π_*θ*_)

The policy π_*θ*_ that determines the action *a*_*t*_ based on the current state *s*_*t*_ is parameterized by the score functions *f*_*κ*_(*x*_*t*_, *c*; *θ*) for *κ* ∈ {tr, rot, tor}. Given state *s*_*t*_, actions are sampled from Gaussian random variables for each degree of freedom, with noise sampled independently for each component:
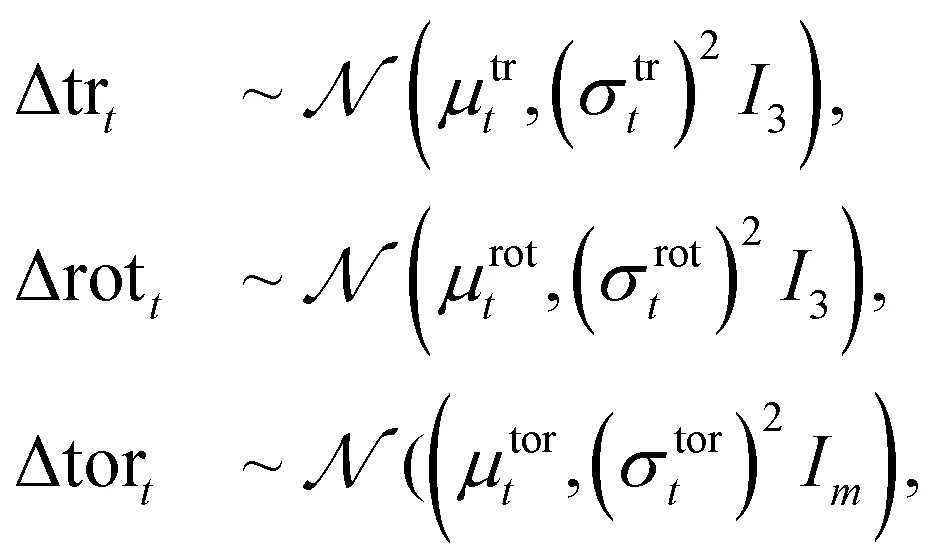
where, for each component *κ* ∈ {tr, rot, tor}, the mean and variance are determined by the score function and diffusion schedule:
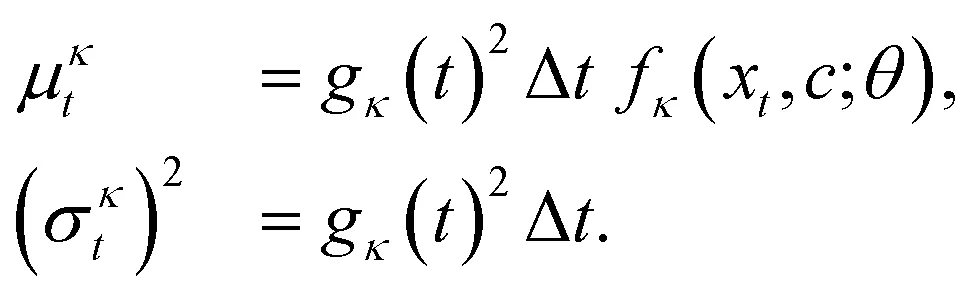


The model thus outputs the mean of the action distribution, while the stochasticity arises from sampling noise according to the diffusion schedule at each timestep. The policy likelihood factorizes across the three separate components with independent noise drawn for each:
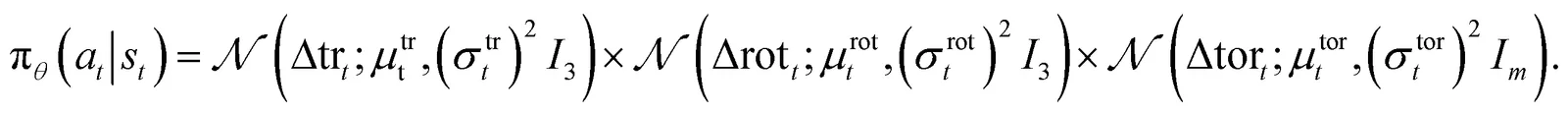


Because the policy is Gaussian with mean determined by the score functions, the log-likelihood of any action taken is tractable and differentiable with respect to *θ*, allowing direct gradient-based optimization of the RL objective.

### 2.3 Policy gradient objective

We optimize the policy using a policy gradient objective with importance sampling. Given a trajectory *τ* = (*s*_*T*_, *a*_*T*_, …, *s*_1_, *a*_1_, *s*_0_) sampled from the policy π_*θ*_old__, where *θ*_old_ denotes the parameters used to sample the trajectory and *θ* the current parameters that are being updated, we normalize rewards across trajectories from the same complex in the batch *via z*-score normalization to obtain a normalized reward *r̃*. Following,^34^ we then optimize the importance-sampled policy-gradient objective
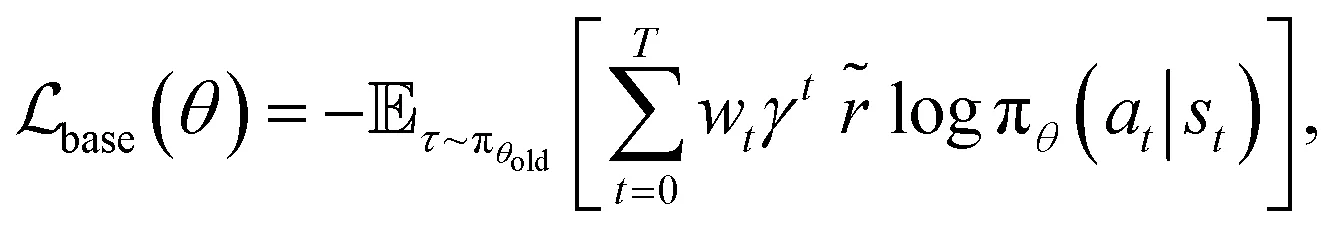
where the per-step importance ratio *ρ*_*t*_(*θ*) = exp(log π_*θ*_(*a*_*t*_|*s*_*t*_) − log π_*θ*_old__(*a*_*t*_|*s*_*t*_)) is clipped symmetrically to prevent excessively large policy updates: *w*_*t*_ = clip(*ρ*_*t*_(*θ*),1 − *∈*,1 +*∈* with *∈* > 0.

### 2.4 Early-step expert guidance

Credit assignment is a central challenge in reinforcement learning,^35^ where a scalar return must be attributed back to a sequence of actions that may contribute unequally to the final outcome. In long-horizon settings with only terminal rewards, standard policy-gradient methods typically propagate a trajectory-level training signal uniformly across all timesteps, which can lead to high-variance updates and weak or misaligned feedback, especially for early actions.

In diffusion-based docking, the credit-assignment problem is exacerbated by the denoising structure of the trajectory. The reward is based solely on the final pose, *x*_0_, yet the policy must choose a sequence of translational, rotational, and torsional updates over many reverse-diffusion steps. An early update that moves the ligand in a favorable direction may still receive poor feedback if later steps degrade the pose, while an early unfavorable update may be rewarded if subsequent denoising recovers a good final structure. As a result, training signals derived solely from terminal outcomes can be poorly aligned with the quality of early decisions.

To mitigate this, we introduce expert guidance for the early, high-noise portion of the trajectory, specifically timesteps *t* ≥ *T*_E_, where *T*_E_ denotes the cutoff in reverse-diffusion time (Fig. 2). Early steps are regularized towards an approximate expert action that steers the ligand towards its (known) ground-truth pose, while later steps are updated using the actions from the sampled trajectory and the terminal reward.

**Fig. 2:**
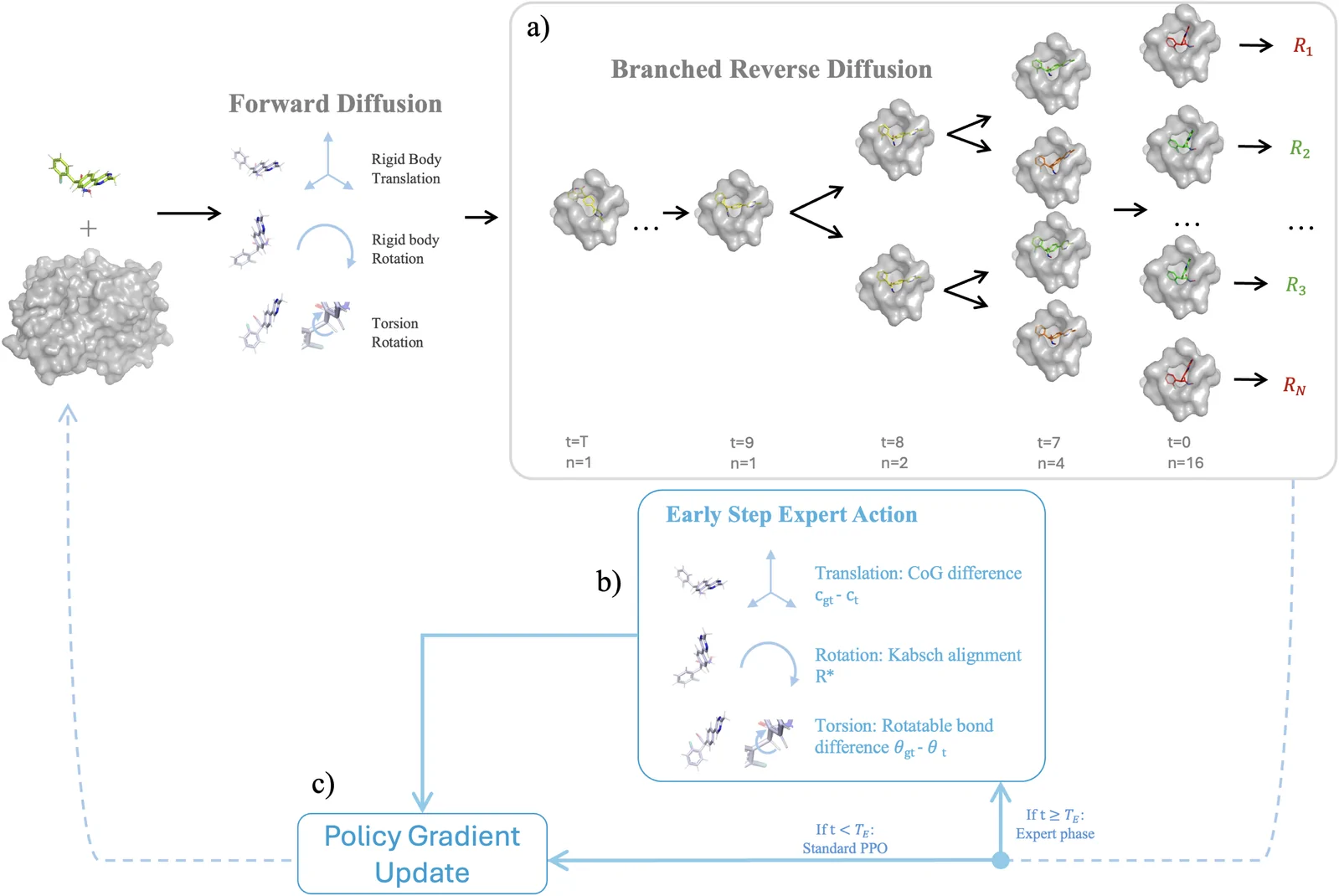
Overview of DiffDock Pocket RL training with early step expert guidance and late step trajectory branching. Starting from a protein ligand complex, Gaussian noise is added to the ligand translation, rotation, and torsion degrees of freedom, before the ligand is denoised through sequential reverse diffusion actions sampled from the policy. (a) During late denoising, trajectories are branched from shared intermediate states to generate multiple terminal poses. Rewards are assigned back through the branching tree, where final unshared actions receive their own terminal reward, shared branching actions receive the mean reward of descendant leaves, and pre branching actions receive the mean reward over all terminal leaves. (b) During expert action timesteps, an approximate expert action is computed from the current ligand state towards the ground truth pose. (c) The policy gradient update is timestep dependent, with early expert action timesteps increasing the likelihood of the calculated expert action, while later reward driven timesteps update the policy using sampled trajectory actions weighted by the assigned terminal or subtree reward.

For each component (translation, rotation, and torsion), we define an approximate optimal action *a*^E^_*t*_ = (Δtr^E^_*t*_,Δrot^E^_*t*_,Δtor^E^_*t*_) that moves the current ligand state toward its experimentally-determined ground-truth pose, scaled by the diffusion step to match the denoising schedule:
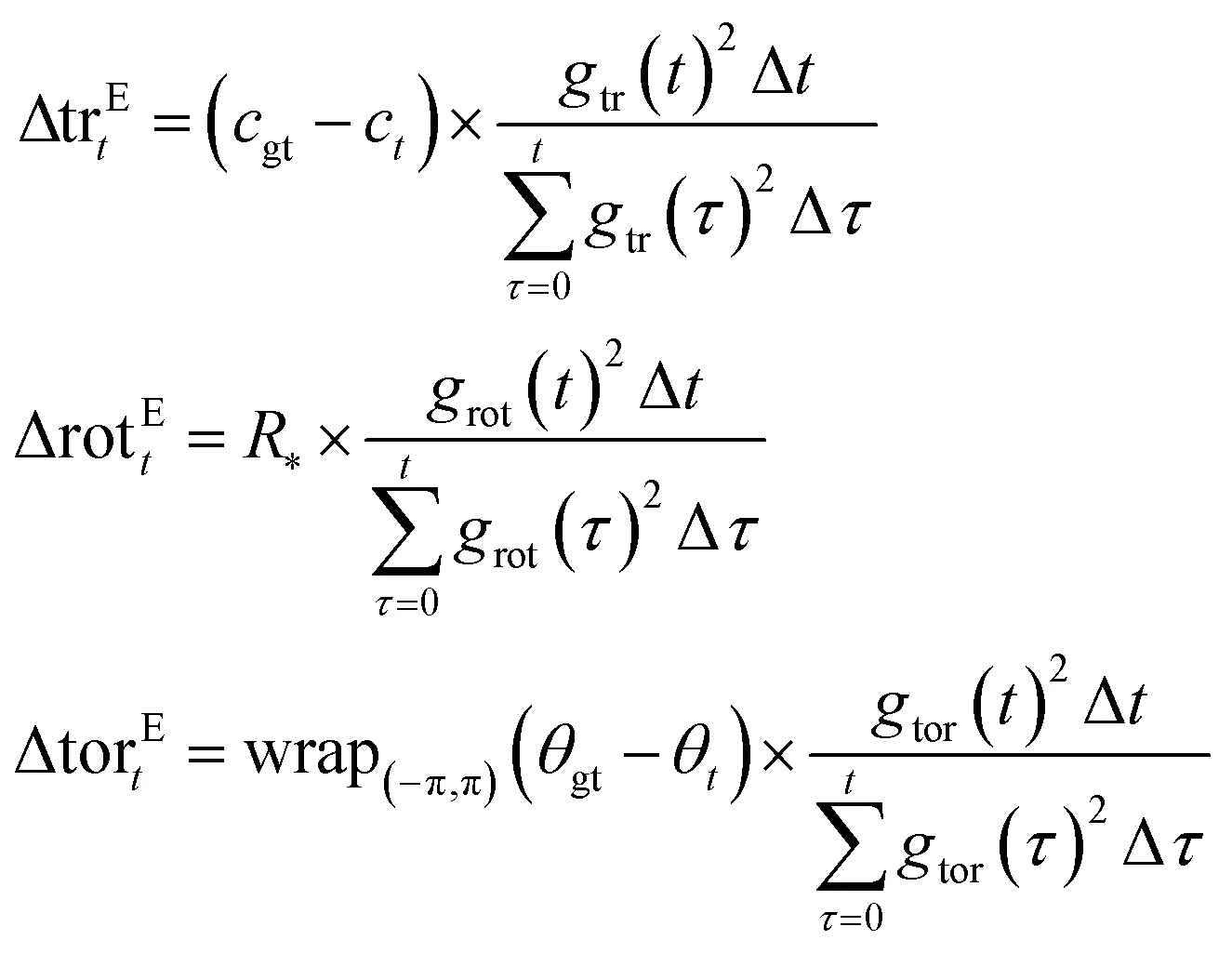
where *c*_*t*_ and *c*_gt_ are the ligand and ground-truth centers of geometry, R* is the optimal rotation from Kabsch alignment, and the torsion differences are wrapped to stay within (−π, π).

We replace the standard DDPO loss with the hybrid objective
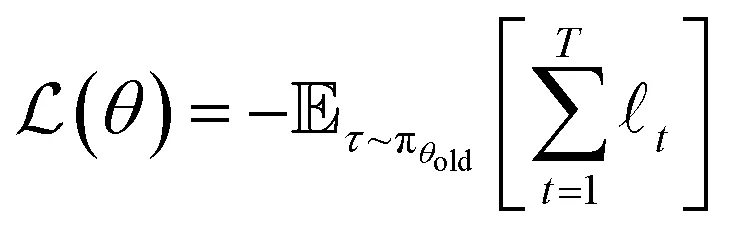
where
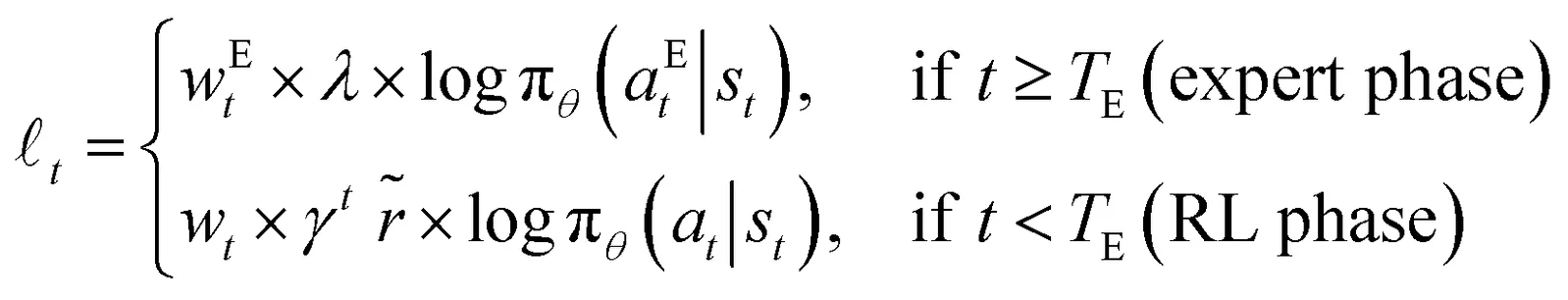
Here, *λ* > 0 is a fixed positive weighting coefficient on the expert log-likelihood term, and *w*^E^_*t*_ is a clipped trust region weight to prevent instability when expert actions lie far outside the current policy distribution.

This expert term is structurally equivalent to a behavior cloning loss applied only to early timesteps, in that it maximizes the likelihood of expert actions *a*^E^_*t*_ under the policy. However, the states *s*_*t*_ are still generated on-policy by π_*θ*_old__, and only updates in the noisy early part of the trajectory are regularized in this way. In practice, the expert phase stabilizes credit assignment for early steps by steering trajectories toward the ground truth, while the RL phase focuses on fine-grained, reward-driven refinement of the final pose.

### 2.5 Late-step trajectory branching

To obtain a denser training signal near the end of the reverse trajectory, where actions are most influential for the final pose and terminal reward, we introduce trajectory branching over a window of late denoising steps (Fig. 3). During this phase, we grow a binary tree of trajectories by resampling noise at each branching timestep from shared intermediate states. Specifically, we branch at each of the four timesteps in the window *t* ∈ {8, …, 5}, with each active trajectory splitting into two descendants at each branching point. This produces 16 leaf poses per complex, all sharing the same early history but diverging progressively over the branching window.

**Fig. 3:**
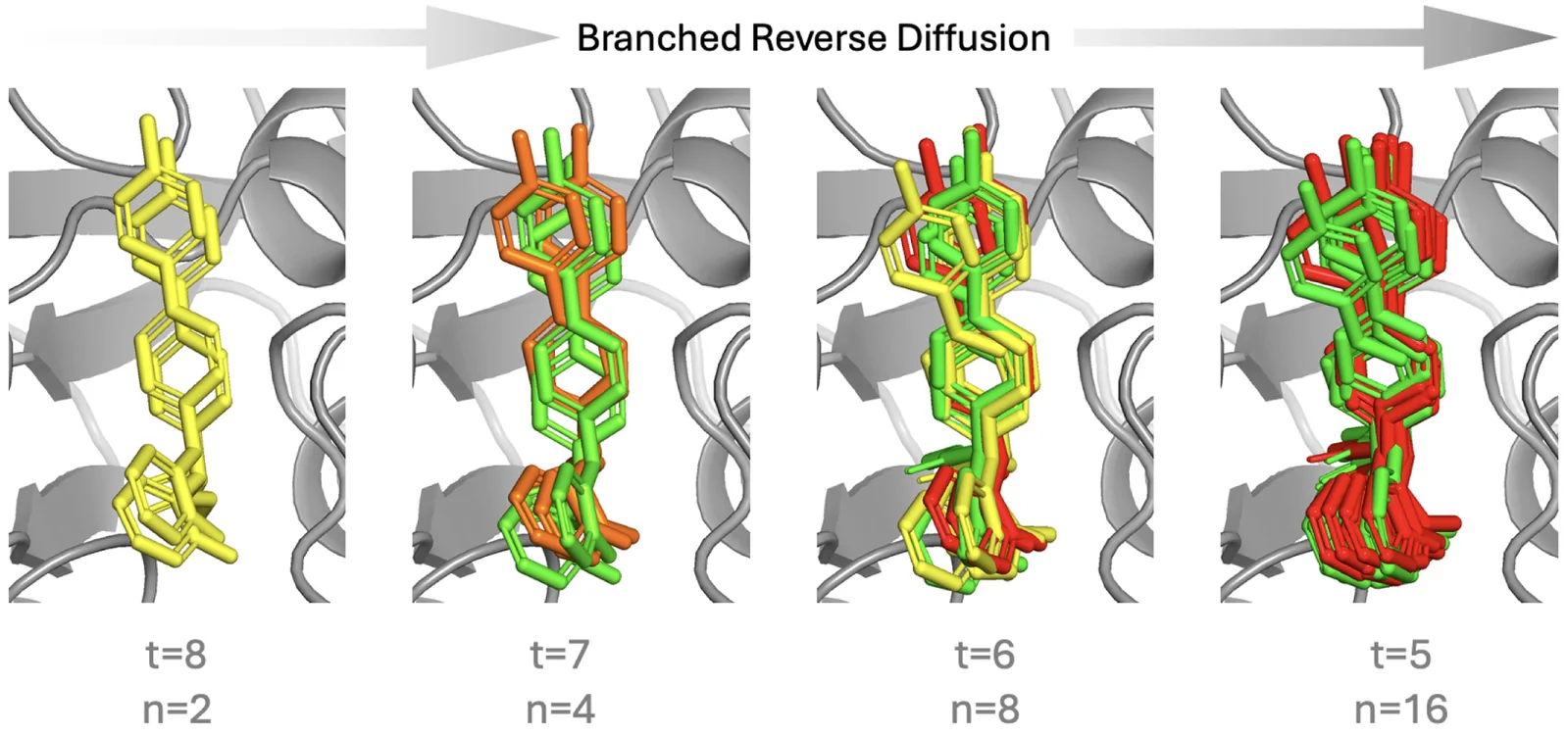
Example of late step trajectory branching during reverse diffusion. Each panel shows n ligand poses in the receptor pocket at successive stages of the branched reverse process. Shared states give rise to multiple branches by resampling noise during late reverse denoising, with ligand colour indicating terminal reward, where green denotes high reward poses, red low reward or invalid poses, and yellow and orange intermediate reward poses.

Each leaf pose 
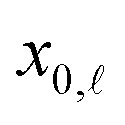
 arises from a distinct sequence of actions during and after the branching window. Because our reward *r*(*x*_0_, *c*) depends on discrete structural and interaction-level criteria, it is a highly non-linear function of the final geometry. Small local perturbations at late denoising steps can therefore induce abrupt changes in reward, for example by introducing or resolving steric clashes or disrupting key contacts. By generating a structured ensemble of end states that progressively diverge from a shared prefix, trajectory branching reveals how these non-linear criteria respond to fine-grained geometric variations at different divergence depths. This provides more informative feedback about which specific refinements turn an almost-valid pose into a valid one, or conversely push a good pose across the decision boundary into an invalid configuration. Since branches share computation up to each branching point, a single branched rollout yields 16 leaf poses without requiring 16 independent trajectory samples.

#### 2.5.1 Reward assignment

Rewards are first normalized within each complex *via* z-score normalization, as described in Section 2.3. Within each rollout, we then use the tree structure to assign per-step rewards. Each internal node *v* at depth *d* (corresponding to branching timestep *t*_d_) is the shared ancestor of a subtree of leaves 
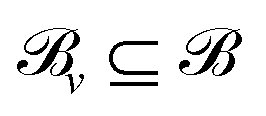
, and receives the mean normalized reward of all its descendants.
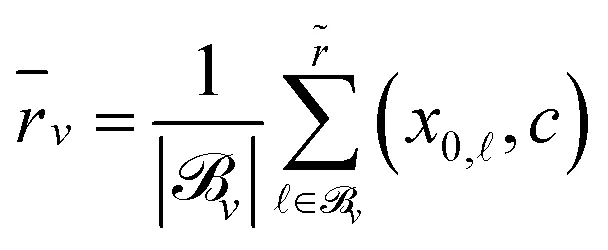


The per-step reward for an action at timestep *t* belonging to leaf trajectory 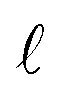 is then
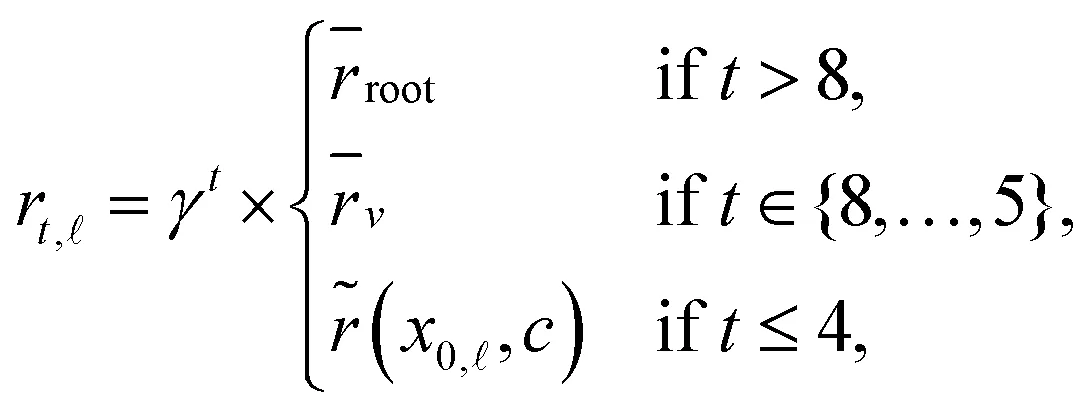
where *v* is the node at depth *d* on the path to leaf trajectory 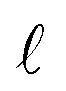, *r̄*_root_ is the mean normalized reward over all 16 leaves in the rollout, and *γ* is the discount factor. Steps before branching begins receive the global rollout mean; steps at each branching timestep in {8, …, 5} receive the mean normalized reward of their descendant subtree; and steps in the final unshared portion of each trajectory *t* ≤ 4 receive that leaf's own normalized reward, decayed by *γ*^*t*^.

This assignment strengthens the learning signal in two ways. First, aggregating rewards across a node's descendant leaves yields a lower-variance estimate of the quality of the policy at that state, improving credit assignment to earlier shared steps. Second, contrasting rewards across branches that differ only in their final denoising steps helps the policy resolve the sharp boundary between physically valid and invalid poses, rather than learning from a single, potentially unrepresentative outcome.

Each branching point thus contributes likelihood terms for all trajectories passing through it, concentrating gradient information at late decision points. In practice, this amplifies the number of gradient updates in late steps and allows greater exploration of small perturbations without requiring multiple independent full trajectories to be generated. We branch over the window *t* ∈ {8, …, 5}, chosen by inspecting generated trajectories and the noise schedule, and observing that meaningful pose variation and the majority of reward-relevant geometric changes occur predominantly in the final steps of the denoising process.

### 2.6 Experimental setup

#### 2.6.1 Reward

We use a terminal reward *r*(*x*_0_, *c*) that rewards both physical validity and predictions that are close to the ground truth. Specifically, we assess the final pose using the PoseBusters physical validity checks and assess whether the generated pose is within 2 Å RMSD of the ground truth structure. The reward is the proportion of all checks passed by the final pose. We use the full set of PoseBusters checks rather than only the most common failure mode, minimum ligand-protein distance, to avoid encouraging reward hacking, for example by moving the ligand away from the protein. Using all checks also maintains all physical validity constraints during training, reducing the risk that the model forgets to pass checks it previously satisfied. After reward normalization, checks that are consistently passed add the same value to every pose, so the learning signal comes from differences between poses rather than from criteria that are already always satisfied. We use a discount factor of *γ* = 0.95. While the PoseBusters package includes a check for whether the RMSD between the generated pose is ≤ 2 Å from the ground truth, we define a structure to be PoseBusters valid (PB-valid) if it passes all of the PoseBusters plausibility checks excluding the RMSD ≤ 2 Å check (*i.e.* all intermolecular, intramolecular, and chemical validity checks that do not require knowledge of the ground-truth pose). Full details of the checks and thresholds are provided in the PoseBusters paper.^30^

#### 2.6.2 Dataset

To train our model, we use the PDBbind 2020 refined set with the same train-validation splits that were used to train DiffDock-Pocket^19^ in order to ensure that any improvement is as a result of our modified training procedure as opposed to a change in data. Complexes whose ground-truth poses fail PB-validity checks are removed, resulting in the exclusion of 562 out of 4503 complexes (12.5%), thereby ensuring clean training signals. Throughout our analysis, we use the PoseBusters benchmark set to assess model performance. This benchmark comprises 308 high-quality protein–ligand complex structures. All entries were deposited in the Protein Data Bank (PDB) after the structures used for training, and sequence similarity metrics are included to assess model generalization.

#### 2.6.3 Evaluation metrics

We evaluate pose quality using four criteria that are generally reported cumulatively as increasingly stringent success definitions: (i) coordinate accuracy, defined as ligand RMSD ≤ 2 Å to the experimental reference; (ii) physical plausibility, defined as passing all PB-validity checks; (iii) interaction recovery (IR) of at least 50%; and (iv) IR of at least 75%. Interaction recovery is calculated using ProLIF as described in,^32^ considering hydrogen bonds, halogen bonds, π-stacking, cation-π, and ionic interactions. Success rates are primarily reported by applying these criteria sequentially. To assess generalizability, we stratify results by sequence identity between the target protein and the most similar protein in the training set, using 0–30%, 30–95%, and 95–100% sequence identity ranges, in line with.^30^ We run DiffDock-Pocket and the fine-tuned DiffDock-Pocket RL for three independent inference runs and report results as mean ± standard deviation across runs. Statistical significance of differences between models is assessed using McNemar's test on per-complex binary success outcomes.

#### 2.6.4 Pose ranking and Top-1 vs. Oracle

Many methods generate multiple candidate poses per complex and use a separate ranking step to select a final prediction. DiffDock-Pocket follows this paradigm, assigning each candidate a score using a separate confidence model. For each complex, we generate 40 candidate poses per method. We report performance using two evaluation protocols: “Top-1”, the highest-ranked pose under the fixed confidence model, and “Oracle”, the best pose among all sampled candidates for that complex according to the evaluation criterion. Thus, Top-1 reflects end-to-end performance including pose selection, while Oracle isolates improvements in the generative model independent of the confidence model. Note we do not modify the confidence model during training.

#### 2.6.5 Comparison to other models

We compare against AutoDock Vina,^10^ GOLD,^12^ and the original DiffDock-Pocket model.^19^ We include Vina and GOLD because physics-inspired docking methods remain a gold standard for generating physically valid poses, providing a strong reference point for plausibility-oriented evaluation. We include the baseline DiffDock-Pocket model to isolate the effect of our reinforcement-learning fine-tuning procedure relative to the same diffusion architecture and inference pipeline. For broader context, we also report the performance for a set of representative ML-based docking methods, namely DiffDock,^18^ EquiBind,^14^ TankBind,^15^ UniMol,^36^ and DeepDock.^37^ We use the same poses for these methods that were used in the original PoseBusters analysis.^30^ Additional implementation details are provided in the PoseBusters paper.^30^

## 3 Results

### 3.1 Physically motivated rewards improve pose validity and plausibility

Our model, trained with RL using the proportion of PoseBusters validity checks passed as a reward, results in a substantial increase in the overall proportion of valid poses generated. Across all complexes, PB-validity increases from 58.8% to 78.1% for the top-ranked pose, and from 38.2% to 58.9% across all sampled poses. A breakdown of performance for each of the PoseBusters criteria for the Baseline DiffDock-Pocket model and RL-fine-tuned DiffDock-Pocket is provided in Section S2. Hereafter, we refer to the RL-fine-tuned model as “DiffDock-Pocket RL” (or sometimes “RL”), and to the original DiffDock-Pocket model as “Baseline”. Fig. 4 shows the proportion of PB-valid poses for the baseline DiffDock-Pocket model and our RL-fine-tuned model stratified by similarity to the training set. We stratify by both sequence identity and, following “Runs N′ Poses”,^38^ by a ligand-pocket similarity metric that accounts for both ligand and binding-pocket similarity. Using RL increases the overall PB-valid fraction across all similarities under both stratification methods. For targets which are the least similar to training set by sequence identity, PB-validity increases from 24.3% to 46.4% for targets with 0–30% sequence identity to the training set. The RL-fine-tuned model also yields substantial improvements for targets closer to the training distribution, increasing PB-validity from 36.4% to 56.8% for targets with 30–95% sequence identity and from 51.5% to 71.2% for targets with 95–100% sequence identity. The ligand-pocket Runs N′ Poses similarity stratification shows the same overall trend with PB-validity increasing from 26.2% to 47.9% in the lowest similarity bin, and DiffDock-Pocket RL remaining consistently higher than the baseline across all similarity bins. These results indicate that directly optimizing PB-validity reliably improves physical plausibility across both in-distribution and out-of-distribution settings, and that this is robust to whether similarity is defined by protein sequence identity or by a combined ligand-pocket similarity metric.

**Fig. 4:**
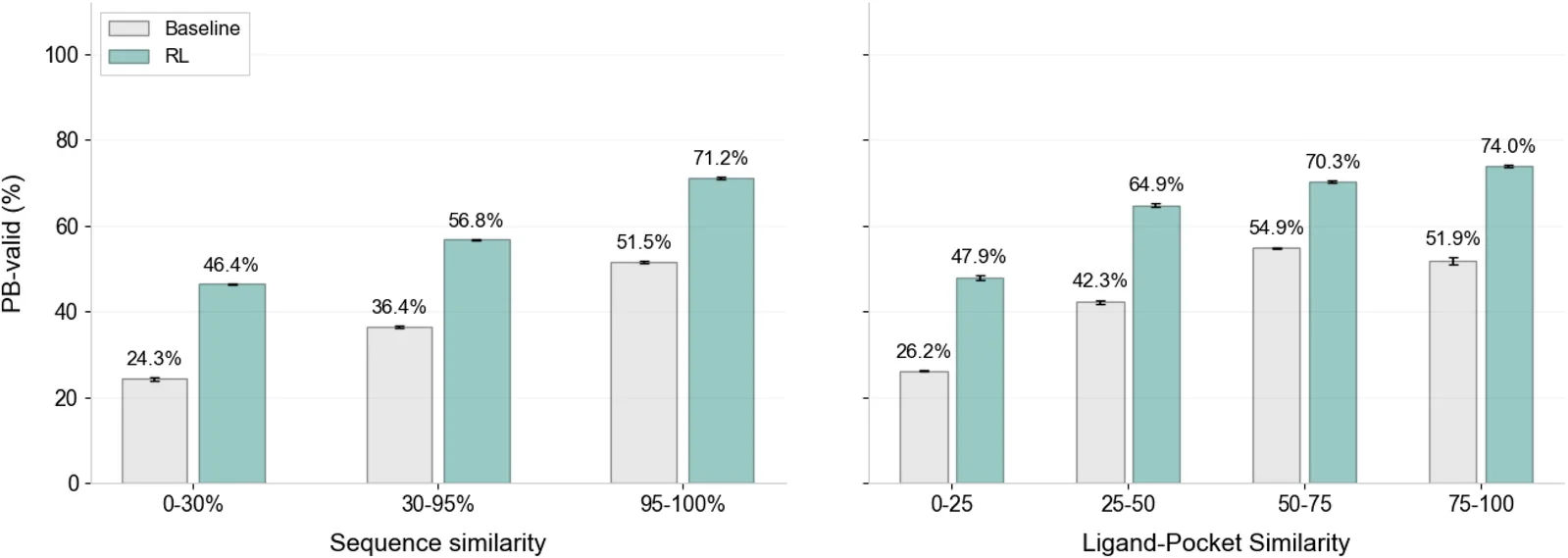
PB-validity of sampled poses stratified by similarity to the training set. PB-validity is shown for the baseline DiffDock-Pocket model and the RL-fine-tuned model under two stratifications: sequence identity to training proteins and the ligand-pocket Runs N′ Poses similarity metric. RL increases the proportion of PB-valid sampled poses across all bins under both stratifications, indicating that RL improves physical plausibility across both in-distribution and out-of-distribution settings.

To evaluate whether our RL-fine-tuned model has improved the quality of docked poses as defined by a physics-based scoring function, we compute Vina energies for every pose generated by both the baseline DiffDock-Pocket model and our RL-trained model. Fig. 5 shows the resulting energy distributions. While Vina energies are an imperfect proxy for binding quality, high energies typically indicate implausible poses, for example due to severe steric clashes, while lower energies suggest greater physical plausibility. Poses generated by the RL model show a substantial shift toward lower energies, decreasing the mean Vina score from 2.24 kcal mol^−1^ to −2.10 kcal mol^−1^, an improvement of 4.34 kcal mol^−1^. This indicates that optimizing for PB-validity both increases physical plausibility directly and also shifts the distribution of generated poses toward lower Vina energies. We additionally compare the Vina energies of the top-ranked RL poses against poses generated directly by Vina and the experimentally observed ligand poses in Section S14.

**Fig. 5:**
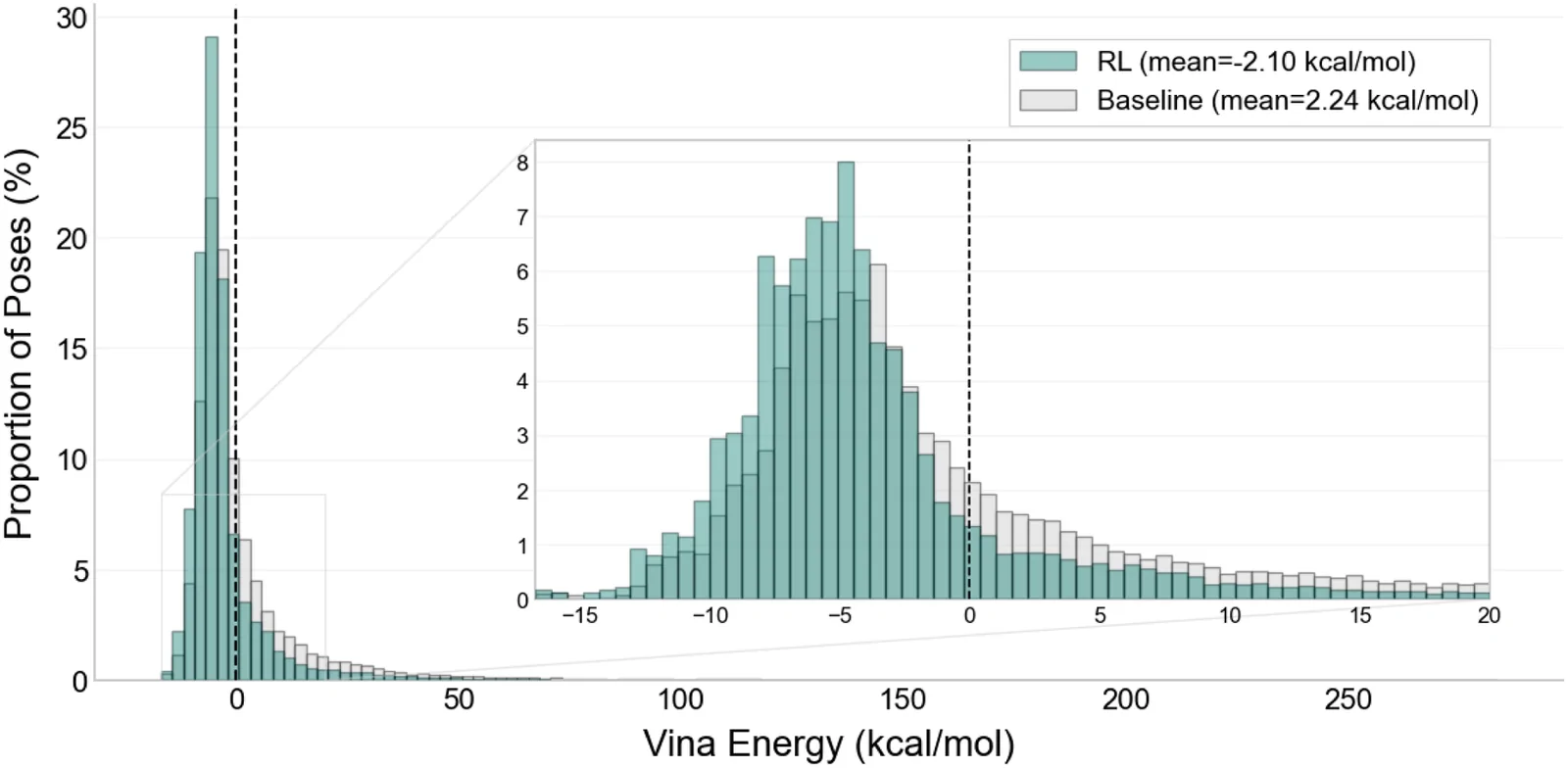
Distribution of Vina energies. Histogram of predicted binding energies (kcal mol^−1^) for poses generated by the baseline model and the RL-optimized model. Reinforcement learning produces a substantial improvement in docking score, decreasing the average Vina energy from 2.24 kcal mol^−1^ to −2.10 kcal mol^−1^.

### 3.2 Reinforcement learning improves performance under all evaluation criteria

Reinforcement learning improves performance across all evaluation criteria, with the most notable gains occurring when physical validity is required (Table 1). Top-1 performance increases from 46.2% to 58.8% when requiring ≤ 2 Å RMSD and PB-valid, and from 38.9% to 49.7% when additionally requiring at least 50% interaction recovery. Substantial improvements are also observed for the Oracle evaluation, which measures whether any generated pose satisfies the criteria regardless of ranking, indicating that RL more reliably generates at least one pose meeting strict combined criteria across complexes. To further assess the ranking model, we analyze the relationship between the DiffDock-Pocket confidence score and pose quality in Section S10. We additionally evaluate docking into predicted apo receptor structures from AFDB in Section S9. Although performance is weaker for both methods than when using experimentally determined holo structures, RL produces similar improvements across the combined physical-validity and interaction-recovery criteria.

**Table 1:** Success rates under increasingly strict quality criteria on the PoseBusters benchmark set (*n* = 308). Our RL fine-tuned version of DiffDock-Pocket (“RL”) improves performance for all criteria compared to the base DiffDock-Pocket model (“Baseline”)^a^

Metric	Top-1 (%)		Oracle (%)	
	Baseline	RL	Baseline	RL
RMSD ≤ 2 Å	66.8 ± 0.5	**69.0 ± 2.8** ^**ns**^	78.0 ± 1.4	84.8 ± 0.4****
& PB-valid	46.2 ± 1.0	58.8 ± 1.7****	66.1 ± 0.7	79.9 ± 0.3****
& ≥ 50% IR	38.9 ± 0.5	49.7 ± 1.7****	61.4 ± 0.9	74.7 ± 0.6****
& ≥ 75% IR	28.5 ± 0.8	35.8 ± 1.8****	50.9 ± 1.0	64.8 ± 1.0****

a Values are reported as mean ± standard deviation across runs. Significance was assessed using McNemar's test on per-complex binary success outcomes, pooled across run pairs. ns: not significant (*p* ≥ 0.05); ****: *p* < 0.0001.

Although our model is trained using only PB-validity as the reward, we also observe improvements in interaction recovery. A plausible explanation is that reducing steric clashes places poses in more plausible contact geometries with the protein, and the increased diversity of valid poses improves the chance of recovering native contacts, which is consistent with our analysis in Section 3.6. The cumulative structure of our evaluation criteria also contributes, as interaction recovery is assessed jointly with physical validity and geometric accuracy, so increases in the number of PB-validity poses increase the pool of poses eligible to satisfy stricter criteria (Table 1).

### 3.3 Reinforcement learning maintains improvement after physics-based relaxation

Previous work has shown that *post hoc* energy minimization can recover some performance lost due to physical implausibility.^30^ To investigate the effect of *post hoc* minimization, we minimized all generated poses using smina^39^ with the Vina scoring function and re-ranked poses using GNINA,^13^ and report Top-1 and Oracle metrics. RL outperforms the baseline across both Top-1 and Oracle metrics, with statistically significant improvements for RMSD ≤ 2 Å and the combined criterion of RMSD ≤ 2 Å and PB-valid (Table 2). For the criteria additionally requiring interaction recovery, Top-1 differences are not statistically significant, though Oracle improvements remain significant, suggesting that while re-ranking does not consistently identify them, minimized RL poses more reliably contain at least one pose meeting strict combined criteria across complexes.

**Table 2:** Success rates under increasingly strict quality criteria on the PoseBusters benchmark set (*n* = 308) after Vina minimization and GNINA re-ranking. Our RL fine-tuned model (“RL”) outperforms the base DiffDock-Pocket model (“Baseline”) across all metrics, showing that improvements persist after *post hoc* minimization^a^

Metric	Top-1 (%)		Oracle (%)	
	Baseline	RL	Baseline	RL
RMSD ≤ 2 Å	77.4 ± 2.1	80.2 ± 1.2*	83.8 ± 1.4	88.5 ± 0.9****
& PB-valid	75.5 ± 1.3	78.2 ± 1.0*	82.9 ± 1.2	87.7 ± 0.9****
& ≥ 50% IR	66.6 ± 1.2	**69.0 ± 1.6** ^ns^	78.9 ± 1.2	82.5 ± 0.3**
& ≥ 75% IR	52.8 ± 1.0	**55.7 ± 1.0** ^ns^	69.2 ± 1.4	73.8 ± 1.0**

a Values are reported as mean ± standard deviation across runs. Significance was assessed using McNemar's test on per-complex binary success outcomes, pooled across run pairs. ns: not significant (*p* ≥ 0.05); *: *p* < 0.05; **: *p* < 0.01; ***: *p* < 0.001; ****: *p* < 0.0001.

Although minimization narrows the gap between models by improving baseline performance relatively more than RL (consistent with the baseline having greater room for improvement due to lower initial physical validity), the RL model maintains an advantage across all Oracle metrics after minimization.

### 3.4 DiffDock-Pocket RL outperforms physics-based and machine learning docking methods

Table 3 compares Top-1 performance for physics-based docking methods and machine learning-based approaches on the PoseBusters benchmark. Prior to physics-based refinement, DiffDock-Pocket RL is already competitive with established physics-based docking methods and outperforms almost all ML-based approaches. DiffDock-Pocket RL achieves 69.0% of predictions with RMSD ≤ 2 Å, outperforming Vina (59.7%) and GOLD (58.1%). When physical validity is additionally required, DiffDock-Pocket RL achieves 58.8% for RMSD ≤ 2 Å and PB-valid, again exceeding Vina (58.1%) and GOLD (54.2%). Under stricter interaction recovery criteria, DiffDock-Pocket RL attains 49.7% for ≥50% IR compared with 44.8% for Vina and 45.5% for GOLD. For the most stringent criterion at ≥75% IR, DiffDock-Pocket RL reaches 35.8%, approaching the best physics-based performance of 37.7% achieved by GOLD.

**Table 3:** Top-1 success rates (%) under increasingly strict quality criteria on the PoseBusters benchmark set (*n* = 308), comparing physics-based and machine learning-based docking methods

Method	RMSD ≤ 2 Å	& PB-valid	& ≥ 50% IR	& ≥ 75% IR
**Physics-based**				
Vina^10^	59.7	58.1	44.8	35.1
GOLD^12^	58.1	54.2	45.5	37.7

**Machine learning-based**				
DeepDock^37^	19.5	5.2	3.2	2.3
DiffDock^18^	38.0	12.3	9.4	7.1
EquiBind^14^	1.9	0.0	0.0	0.0
TankBind^15^	15.9	3.9	1.9	1.3
UniMol^36^	20.8	1.9	0.6	0.6
DiffDock-Pocket^19^	66.8	46.2	38.9	28.5
GNINA^a^^13^	73.7	72.4	56.2	40.9
DiffDock-Pocket RL	69.0	58.8	49.7	35.8
DiffDock-Pocket RL++	**80.2**	**78.2**	**69.0**	**55.7**

a GNINA combines physics-based search with a machine learning-based scoring function to rank poses.

As shown in Section 3.3, the performance of DiffDock-Pocket RL can be improved by minimizing poses using Vina and re-ranking using GNINA at minimal additional computational cost, in part by replacing the original DiffDock-Pocket confidence model. This approach, denoted “DiffDock-Pocket RL++”, achieves the best performance of all methods tested, with 80.2% of predictions with RMSD ≤ 2 Å and 78.2% when PB-valid is additionally required. Under stricter interaction recovery thresholds, performance reaches 69.0% for ≥50% IR and 55.7% for ≥75% IR. These results surpass both physics-based and machine learning-based docking approaches with comparable binding site definitions. The improvement over other ML methods is particularly substantial when physical validity is required, with DiffDock-Pocket RL++ achieving 78.2% for RMSD ≤ 2 Å and PB-valid, compared with 72.4% for GNINA, 46.2% for DiffDock-Pocket, 12.3% for DiffDock, and below 10% for all other ML baselines. Overall, this demonstrates that reinforcement learning can effectively enhance diffusion-based docking models, and that combining learned generative models with physics-based refinement yields strong docking performance. For completeness, we also report additional comparisons in Section S1, including results for machine-learning docking methods alongside visualisations of their corresponding pocket definitions. We also include a comparison to AlphaFold3 with an accompanying training-set similarity analysis in Section S12.

### 3.5 Early-step guidance and late-step branching ablation

We next assess the contribution of early-step expert guidance and late-step trajectory branching on model performance. We compare DiffDock-Pocket RL against Baseline and ablated RL models trained without early guidance, without branching, or without either component. We evaluate each method on the PoseBusters benchmark using the joint success criterion RMSD ≤ 2 Å and PB-validity, which directly measures whether a generated pose is both near-native and physically plausible.

As shown in Table 4, the Top-1 results show that both late-step trajectory branching and early-step guidance contribute to performance. Removing branching causes the largest reduction, decreasing Top-1 success from 58.8% to 49.6%. Removing early-step guidance produces a smaller but still statistically significant decrease to 54.7%, while removing both components gives the weakest RL-trained variant at 49.1%. However, all RL-trained variants still outperform DiffDock-Pocket, which achieves 46.2%. Similar trends are observed for Oracle performance. Overall, the strongest performance is obtained when both components are used together.

**Table 4:** Success rates for ablation models on the PoseBusters benchmark set (*n* = 308). DiffDock-Pocket RL, trained with both early-step guidance and late-step trajectory branching, outperforms DiffDock-Pocket and the ablated RL variants for the joint criterion RMSD ≤ 2 Å and PB-validity^a^

Method	Top-1 (%)	Oracle (%)
DiffDock-Pocket RL	**58.8 ± 1.7**	**79.9 ± 0.3**
No branching	49.6 ± 1.8****	70.0 ± 0.5****
No early guidance	54.7 ± 2.1**	79.7 ± 1.2^ns^
No branching or guidance	49.1 ± 3.4****	69.4 ± 1.0****
DiffDock-Pocket	46.2 ± 1.0****	66.1 ± 0.7****

a Values are reported as mean ± standard deviation across three runs. Superscripts indicate significance against DiffDock-Pocket RL, assessed using McNemar's test on paired per-complex binary success outcomes pooled across matched run pairs. ns: not significant (*p* ≥ 0.05); *: *p* < 0.05; **: *p* < 0.01; ***: *p* < 0.001; ****: *p* < 0.0001.

### 3.6 Analysis of generated poses

In this section, we analyze the poses generated by DiffDock-Pocket RL and the original DiffDock-Pocket model to understand the reason for the observed performance differences. This analysis reveals how RL training changes the distribution of generated poses and identifies the failure modes it corrects.

Fig. 6 shows the distribution of average pairwise RMSD between all valid poses for each complex, examining whether the RL model generates diverse physically valid poses or reproduces the same valid pose many times for a given complex. RL exhibits higher mean pairwise RMSD (2.21 Å) relative to the baseline DiffDock-Pocket model (1.25 Å) alongside more complexes with PB-valid poses (291 *vs.* 244). The simultaneous increase in diversity and validity, alongside the disproportionate increase in physically valid poses observed for low-homology targets, suggests the model has learned generalizable principles of validity rather than relying on memorization of valid training poses.

**Fig. 6:**
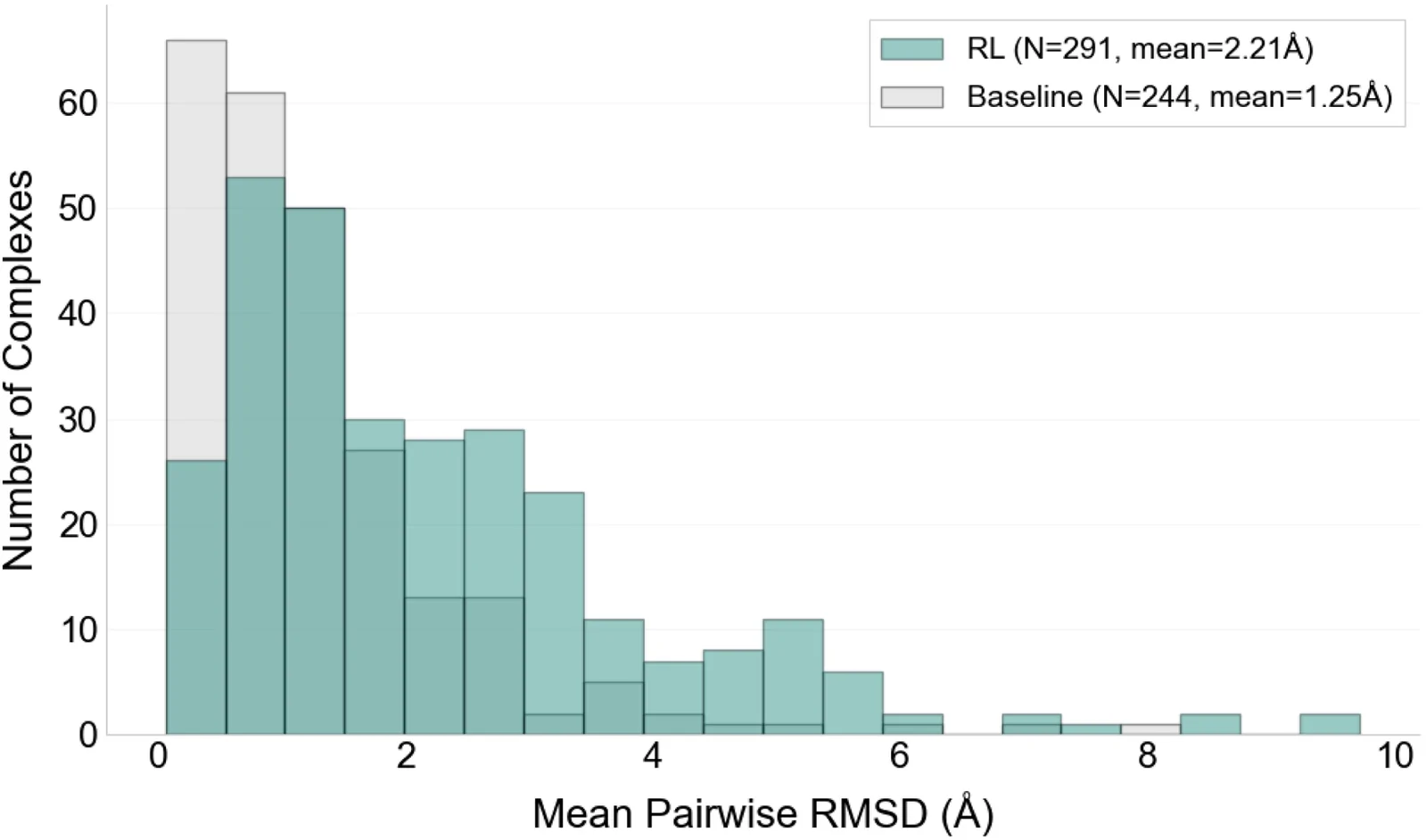
Diversity of PB-valid poses. Distribution of average pairwise RMSD between PB-valid poses within the full generated set for each of the PoseBusters benchmark complexes (*N* = 308). The RL model generates more diverse valid poses (mean pairwise RMSD 2.21 Å) than the baseline (1.25 Å).

We analyze the failure modes of the original DiffDock-Pocket (“Baseline”) on cases where RL succeeded and summarize the results in Table 5. We ran each model three times, generating 40 poses per complex per run. For each of the three runs, we identify complexes for which RL produced at least one pose that is both PB-valid and ≤ 2 Å RMSD, while the baseline produced no such pose across all 40 generated poses. This yields 139 such cases across the three runs, corresponding to 69 unique complexes. For these cases, we classify the failure modes of the baseline model according to whether it produced PB-invalid poses with ≤ 2 Å RMSD, PB-valid poses with > 2 Å RMSD, PB-invalid poses with > 2 Å RMSD, or both ≤ 2 Å RMSD poses and PB-valid poses but never simultaneously. We also report the best RMSD achieved across all baseline poses and, if any were generated, the best RMSD among PB-valid baseline poses. Section S11 also provides Venn diagrams summarizing the overlap in success sets between the original DiffDock-Pocket baseline and the RL-trained DiffDock-Pocket model, stratified by sequence similarity.

**Table 5:** Failure mode analysis of the baseline DiffDock-Pocket model on cases where RL succeeds (69 unique complexes, 139 complex-run pairs). The majority of baseline failures occur because near-native poses are not PB-valid (38.1%), though RL also succeeds in many cases where the baseline fails to reach the native binding mode entirely

Failure mode	Count (%)	Best RMSD overall	Best RMSD among valid
RMSD ≤ 2 Å and not PB-valid	53 (38.1%)	1.22 ± 0.46 Å	N/A
RMSD > 2 Å and PB-valid	42 (30.2%)	3.57 ± 1.86 Å	4.71 ± 2.48 Å
RMSD > 2 Å and not PB-valid	22 (15.8%)	4.07 ± 1.88 Å	N/A
RMSD ≤ 2 Å or PB-valid separately	22 (15.8%)	1.28 ± 0.37 Å	5.54 ± 3.74 Å

Table 5 shows that, in most cases where the baseline model fails to produce a pose that is both PB-valid and ≤ 2 Å RMSD, the failure occurs because near-native poses are not PB-valid. In 38.1% of cases, the baseline achieves ≤ 2 Å RMSD but no PB-valid poses are generated. However, RL also succeeds in many cases where the baseline does not reach the native binding mode. In 30.2% of cases, the baseline produces PB-valid poses but none ≤ 2 Å RMSD, with a mean best RMSD among valid poses of 4.71 Å, suggesting that it samples physically plausible poses with an alternative binding mode. Furthermore, the mean best RMSD achieved across all poses for these cases is 3.57 Å, indicating that the baseline is not close to the native binding mode even among its invalid poses. Finally, 15.8% of cases contain no PB-valid poses and no poses ≤ 2 Å RMSD across all sampled poses, and a further 15.8% of cases contain both a PB-valid pose and a ≤ 2 Å pose but never simultaneously, indicating that the baseline is often unable to generate poses that are both near native and valid where RL does.

## 4 Conclusions

In this work, we demonstrate that reinforcement learning can optimize diffusion-based docking models for non-differentiable objectives, teaching them to generate a higher proportion of physically valid molecular poses without sacrificing structural accuracy. These improvements arise solely from updating the score-model weights resulting in no increase in inference-time compute relative to the base model. Fine-tuning DiffDock-Pocket using a non-differentiable terminal reward based on PB-validity improves Top-1 success for poses that are both ≤ 2 Å RMSD and PB-valid from 46.2% to 58.8%, and Oracle success from 66.1% to 79.9%. The most substantial improvements are observed in the lowest-homology targets (0–30% sequence identity), suggesting that the RL-based training has improved generalization.

Our primary aim was to improve the quality of the generated poses, and thus in this work we did not seek to improve or retrain the ranking model used by DiffDock-Pocket, despite the shift in pose distribution as a result of RL training. As a consequence, the improvement in Oracle performance was larger than Top-1, suggesting limitations in the ranking approach adopted. We show that minimizing poses generated by our approach using a physics-based method and re-ranking can yield substantial improvements, and recommend this approach be used in practice. DiffDock-Pocket RL++ achieves 80.2% Top-1 success for RMSD ≤ 2 Å and 78.2% when physical validity is additionally required, surpassing all physics-based and ML-based docking methods tested on the PoseBusters benchmark. Improving the confidence model is a promising direction for future work and could result in more of the improvement in Oracle performance being translated into Top-1 success.

While recent work has begun to explore guidance-based approaches to improve pose validity in other applications of diffusion models in SBDD, such as Boltz-steering,^29^ these methods require additional forward passes and validity checks during denoising, increasing memory use and inference time. More fundamentally, guidance reshapes sampling at inference time without changing the model, so it is not expected to lead to improvements when the base model places the ligand in the wrong region of the target. Our reinforcement learning framework instead updates the learned score function so that the model itself assigns higher probability to physically valid poses and to poses that are both valid and near-native. Although our method yields substantial improvements over DiffDock-Pocket in the low sequence similarity regime, performance remains lower on the most dissimilar targets than on those closer to the training distribution. Closing the gap on the most out of distribution targets therefore remains an open challenge.

Recent cofolding tools, such as AlphaFold3 and Boltz,^26,29^ and diffusion-based molecular generation models, such as DiffSBDD,^27^ have been shown to suffer similar failure modes, such as generating physically invalid structures,^31^ suggesting both could benefit from RL fine-tuning. By using non-differentiable physical criteria as training signals, we can optimize for constraints that are difficult to encode through supervised losses alone. As the field continues to develop increasingly powerful generative models for biomolecular structures, ensuring their outputs are not just accurate but also physically valid and useful for downstream screening and lead optimization will be essential for translating these advances into practical tools for drug discovery and protein engineering.

We show that diffusion-based docking models can be trained to more explicitly respect physical principles that are not captured by the standard supervised objective. Reinforcement learning enables these constraints to be incorporated directly into training through non-differentiable objectives while maintaining structural accuracy. This provides a general approach for improving the physical validity of predictions made by diffusion-based models for structure prediction and can be extended to other structure-based prediction tasks.

## Author contributions

J. H. B., F. I., C. M. D., and B. P. conceived the study. J. H. B. and F. I. developed the methodology. J.H.B. developed the software. F. I., C. M. D., B. P., and D. K. supervised the work. J. H. B. and F. I. wrote the initial draft of the manuscript. All authors reviewed and edited the manuscript and approved the final version.

## Conflicts of interest

There are no conflicts to declare.

## Supplementary Material

SC-017-D6SC02655A-s001

## Data Availability

Training and inference code is available at https://github.com/oxpig/RLDiff under the MIT License. The PoseBusters Benchmark Set complexes used in this study are available from Zenodo at https://zenodo.org/records/8278563. The PDBbind v2020 set used for training is available from PDBbind+ at https://pdbbind-plus.org.cn/download to registered users at no cost. Supplementary information (SI) is available. See DOI: https://doi.org/10.1039/d6sc02655a.
