# Supplementary material for: Teaching diffusion models physics: reinforcement learning for physically valid diffusion-based docking
Source: Chem Sci. 2026 Aug 18;17(40):19447–58. doi: 10.1039/d6sc02655a (PMC13531412; doi:10.1039/d6sc02655a)
Supplement: Supplementary file 1 [file SC-017-D6SC02655A-s001.pdf]

Supplementary Information (SI) for Chemical Science.
This journal is © The Royal Society of Chemistry 2026

Supplementary Information

Teaching Diffusion Models Physics: Reinforcement Learning for Physically Valid Diffusion-Based Docking

J. Henry Broster[1], Bojana Popovic[2], Diana Kondinskaia[2], Charlotte M. Deane[1], and Fergus Imrie[1]

[1]Department of Statistics, University of Oxford, Oxford, OX1 3LB, United Kingdom
[2]Cambridge Crystallographic Data Centre, Cambridge, CB2 1EZ, United Kingdom

This document provides supplementary figures, tables, and analyses supporting the manuscript "Teaching Diffusion Models Physics: Reinforcement Learning for Physically Valid Diffusion-Based Docking".

Contents

S1 Pocket Definitions Across Docking Methods **2**
S2 PoseBusters Physical Validity Checks **3**
S3 PoseBusters Validity Checks Stratified by Sequence Identity **4**
S4 Sample Efficiency **5**
S5 PB-Validity Stratified by RMSD to Crystal Pose **6**
S6 Per-Complex PB-Valid Pose Comparison **7**
S7 Stratified Results by Sequence Similarity to the Training Set **8**
S7.1 Minimized Structures . . . 8
S8 Stratified Results by PDBbind SuCOS-Pocket Similarity **9**
S9 Results for Docking into Apo Structures **10**
S9.1 Stratified Apo Results by Sequence Similarity to the Training Set . . . 11
S9.2 Minimized Apo Structures . . . 12
S10 Confidence Score and Pose Quality **13**
S11 Overlap in Success Sets Across Sequence Identity Groups **15**
S12 Comparison to AlphaFold3 **17**
S13 Training Hyperparameters **18**
S14 Comparison with Vina-Generated and Experimentally Observed Poses **19**

S1 Pocket Definitions Across Docking Methods

	**Vina**[1]	**GOLD**[2]	**DiffDock-Pocket**[3]
$\leq$ 2 Å RMSD	59.7	58.1	66.8
& valid	58.1	54.2	46.2

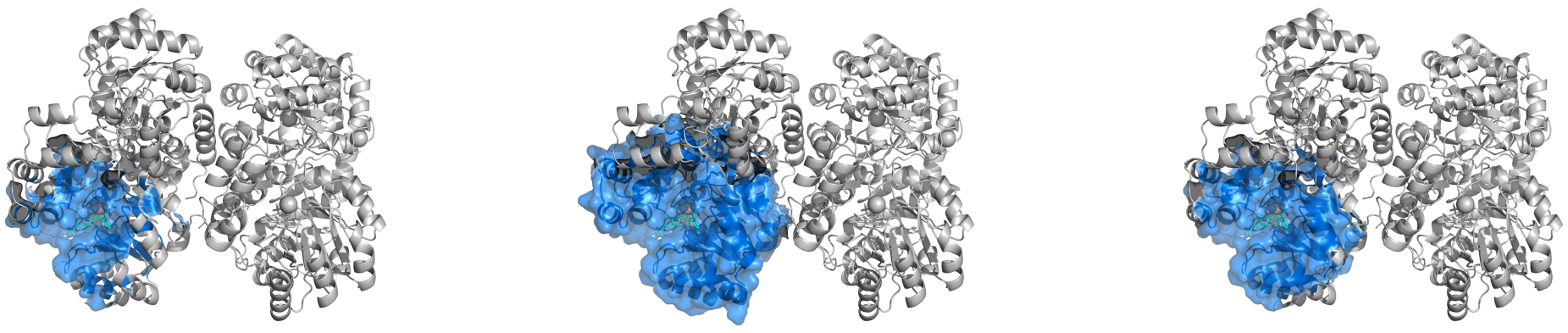

	**SigmaDock**[4]	**UniMol**[5]	**DeepDock**[6]
$\leq$ 2 Å RMSD	69.8	20.8	20.0
& valid	68.2	1.9	2.0

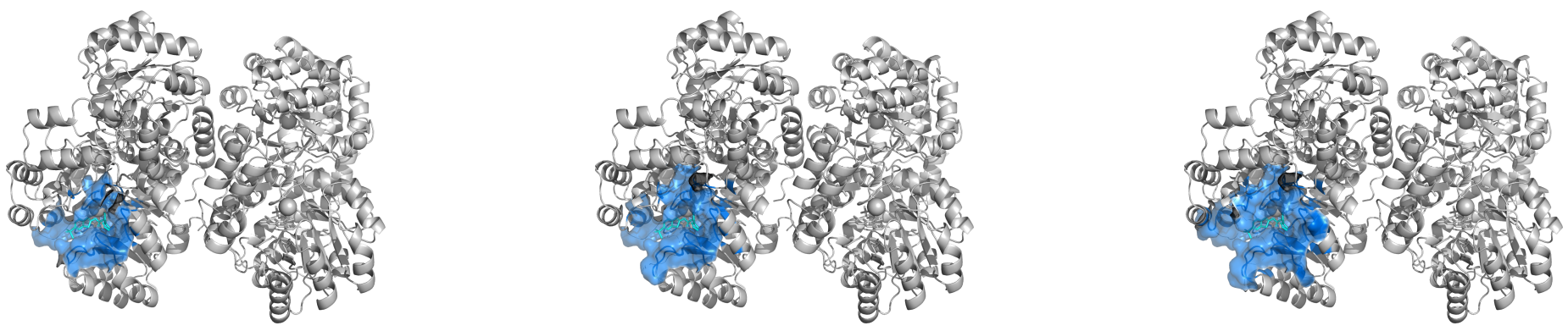

Fig. S1: Pocket definitions and performance across docking methods. The figure shows performance alongside visualisations of the corresponding pocket definitions.

The binding sites into which ligands are docked are defined as follows:

AutoDock Vina[1]**:** The search space is defined as a cubic bounding box of side length 25 Å centred at the ligand centroid.

GOLD[2]**:** The binding site is defined as a sphere of radius 25 Å centred at the ligand centroid.

DiffDock-Pocket[3]**:** Receptor Cα atoms within 5 Å of ligand heavy atoms are selected and their mean coordinate defines the pocket centre. The pocket radius is set as the maximum distance from this centre to any ligand heavy atom plus a 10 Å buffer.

SigmaDock[4]**:** All receptor residues within a distance threshold d_0 of any ligand heavy atom are included. We report $d_0 = 7$ Å for greatest comparability with other approaches.

UniMol[5]**:** All receptor residues with any atom within 8 Å of a ligand heavy atom are included.

DeepDock[6]**:** The binding region is defined as a surface patch extending approximately 10 Å from the ligand.

S2 PoseBusters Physical Validity Checks

Figures S2 and S3 provide a breakdown of pass rates for each individual PoseBusters validity test. Figure S2 shows results for the top-ranked pose per complex, while Figure S3 aggregates across all 40 sampled poses. The largest improvements from RL training occur in the "Minimum Distance To Protein" test, improving from 60.4% to 80.3% for top-ranked poses. "Volume Overlap With Protein" also improves from 95.5% to 99.2%, indicating that RL substantially reduces steric clashes. Improvements are also observed for interaction geometry tests involving cofactors and water molecules.

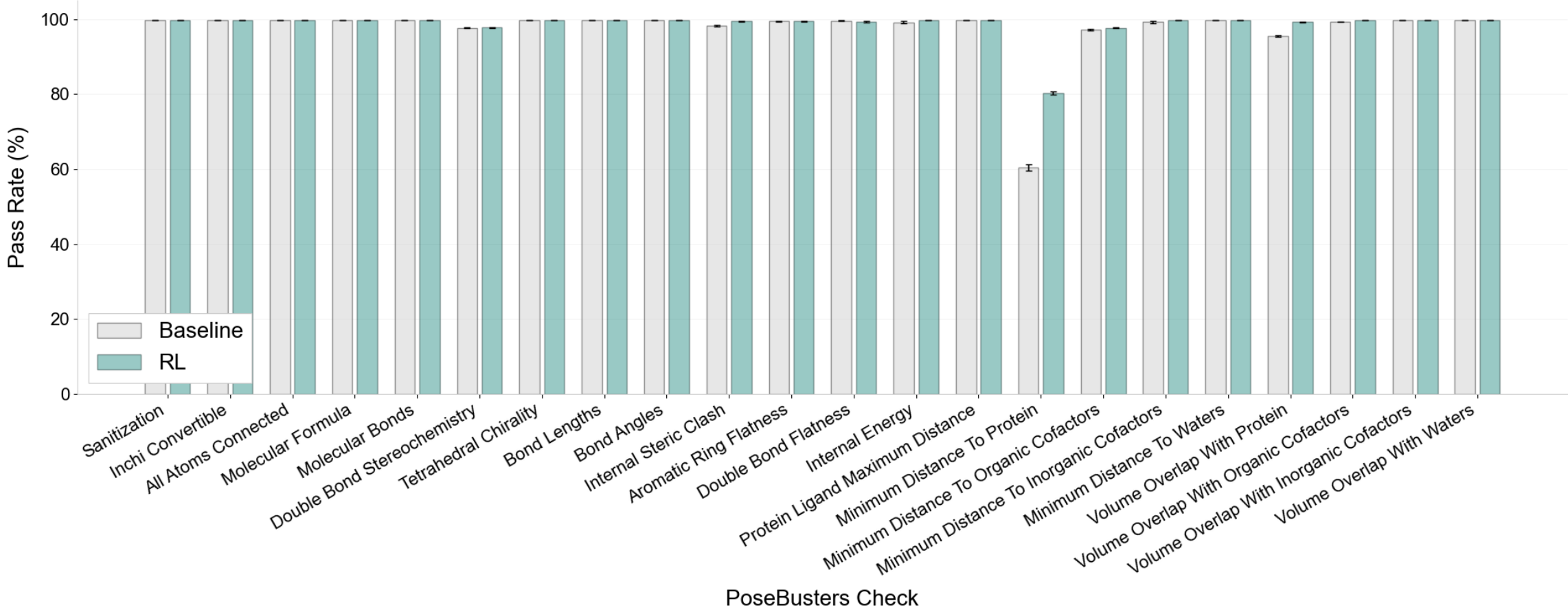

Fig. S2: Pass rates for individual PoseBusters validity tests on top-ranked poses. Baseline model (gray) compared against RL-trained model (blue). Error bars show standard error across three independent runs.

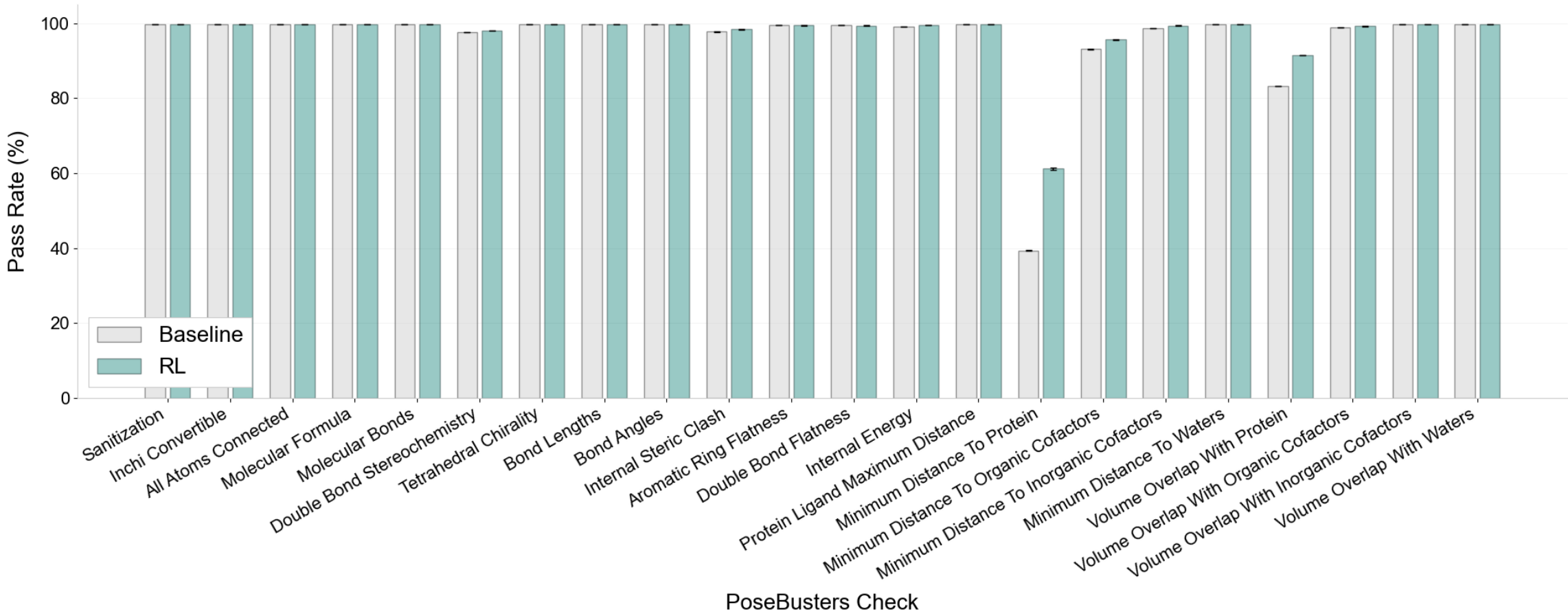

Fig. S3: Pass rates for individual PoseBusters validity tests aggregated across all 40 sampled poses per complex. Baseline model (gray) compared against RL-trained model (blue). Error bars show standard error across three independent runs.

S3 PoseBusters Validity Checks Stratified by Sequence Identity

Figures S4–S6 show pass rates for individual PoseBusters validity tests stratified by sequence identity to the training set, aggregated across all poses. RL training provides the largest improvements for low-homology targets (0–30% sequence identity), where "Minimum Distance To Protein" improves from 25.3% to 48.8% (a 92.9% relative improvement), and "Volume Overlap With Protein" improves from 74.4% to 86.8%. For high-homology targets (95–100%), RL training further improves "Minimum Distance To Protein" from 53.0% to 73.5%, demonstrating consistent benefits across different sequence similarities.

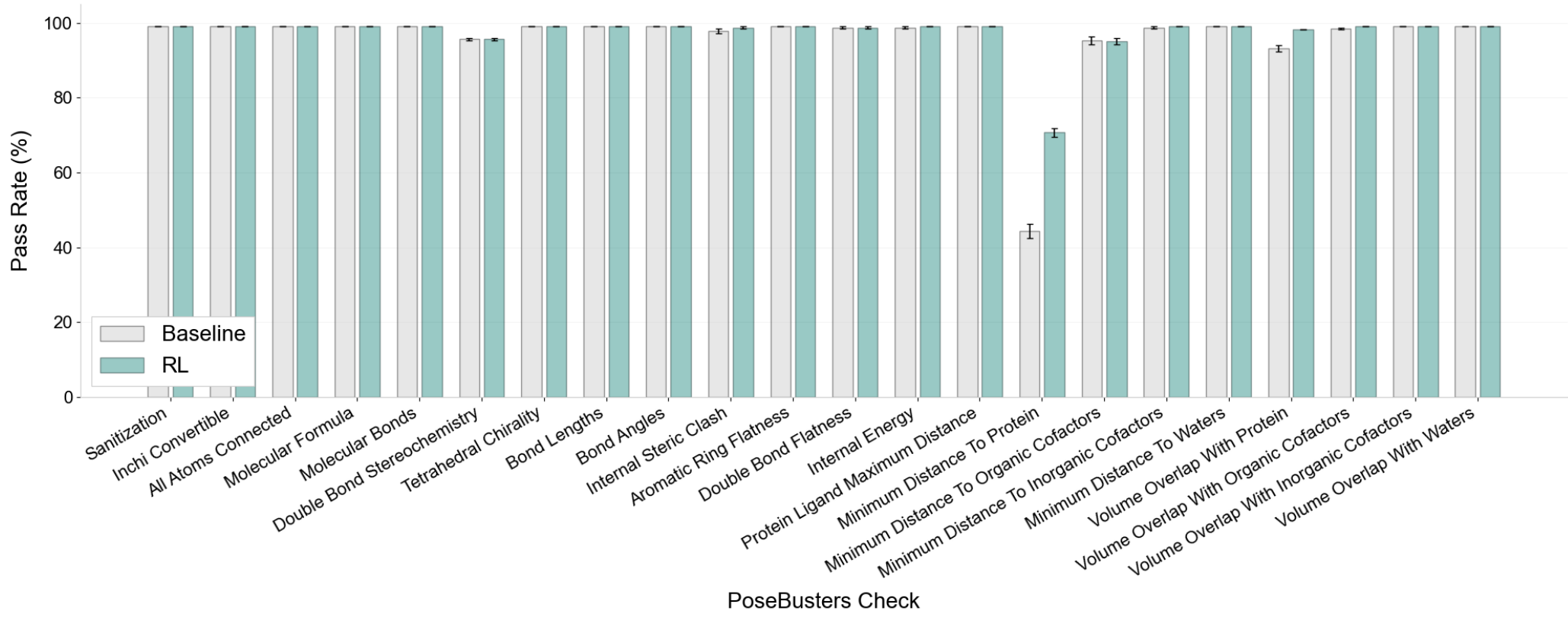

Fig. S4: Success rates for PoseBusters checks (0-30% sequence identity).

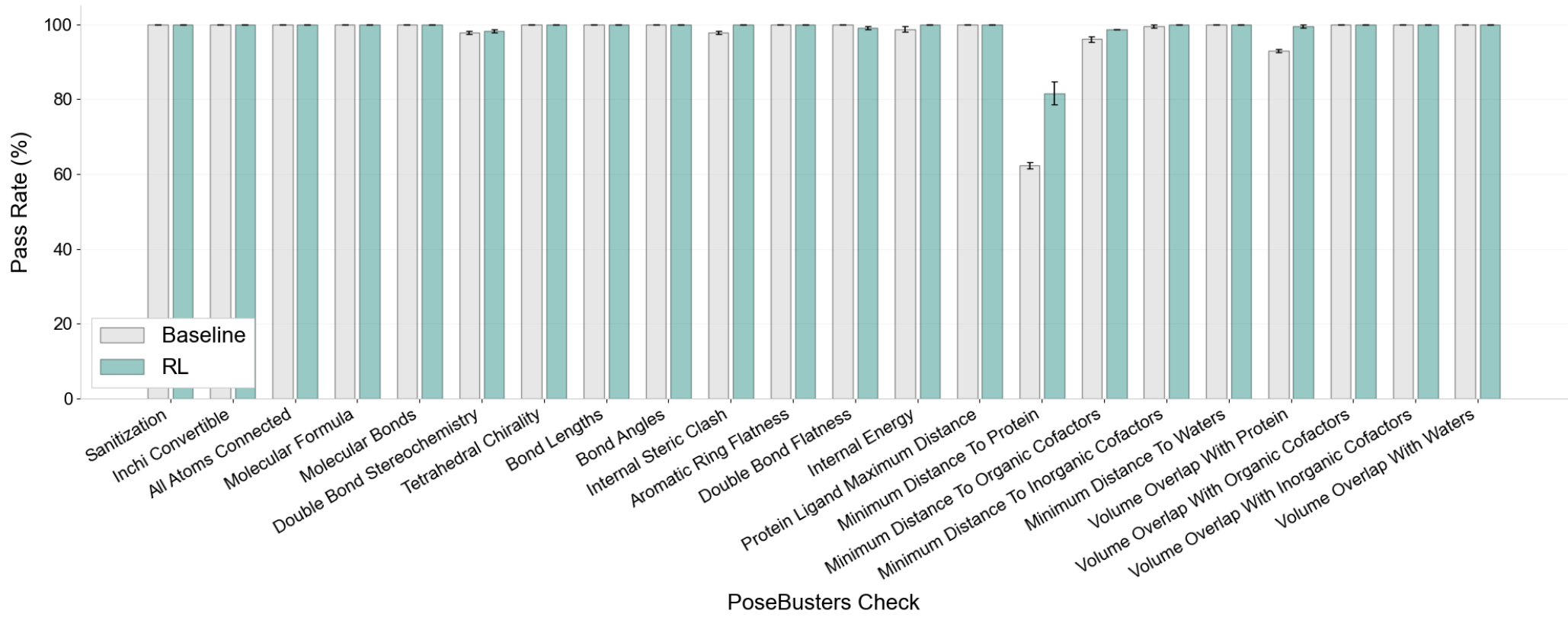

Fig. S5: Success rates for PoseBusters checks (30-95% sequence identity).

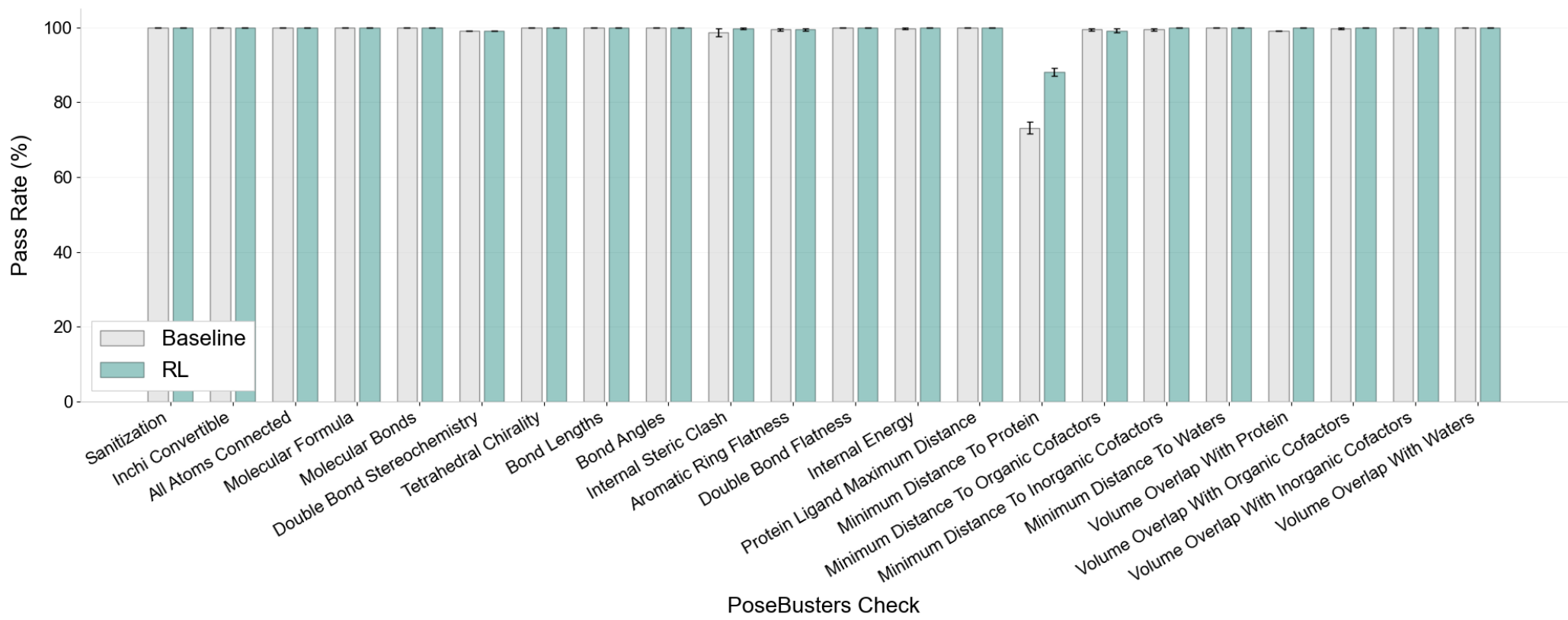

Fig. S6: Success rates for PoseBusters checks (95-100% sequence identity).

S4 Sample Efficiency

Figure S7 shows success rates for the joint criterion RMSD ≤ 2 Å and PB-valid as a function of the number of poses sampled per complex, for both Top-1 and Oracle evaluation. Under Top-1, RL consistently outperforms the baseline across all sample sizes. Under Oracle evaluation, the gap between RL and the baseline widens substantially with more samples, indicating that RL generates a richer ensemble of valid poses and benefits more from additional sampling.

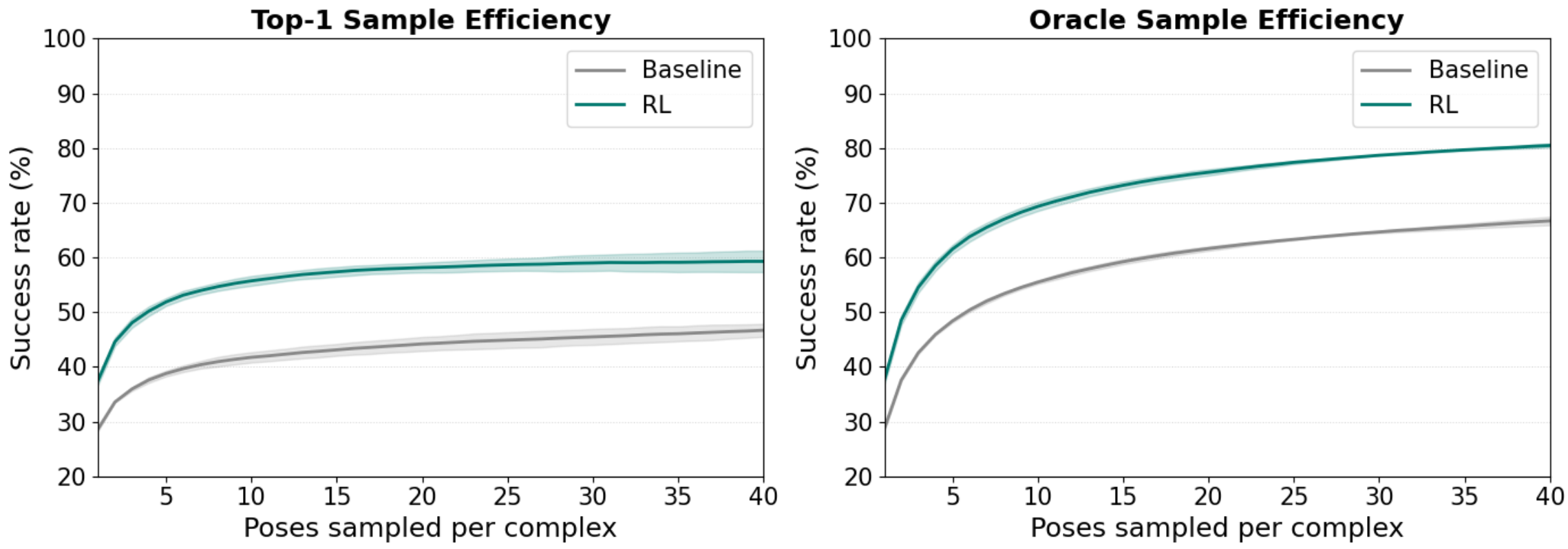

Fig. S7: Sample efficiency curves showing success rate (RMSD ≤ 2 Å and PB-valid) as a function of poses sampled per complex. Shaded regions show standard error across runs.

S5 PB-Validity Stratified by RMSD to Crystal Pose

Figure S8 shows the proportion of PB-valid poses stratified by whether the pose is within 2 Å RMSD of the crystal structure. For near-native poses (RMSD $\leq$ 2 Å), RL increases PB-validity from 56.6% to 79.7%, demonstrating that RL substantially improves the physical plausibility of poses that are already close to the ground truth. For poses further from the crystal structure (RMSD $>$ 2 Å), PB-validity also improves from 19.9% to 40.7%. This confirms that RL does not simply trade geometric accuracy for physical validity; rather, it improves physical plausibility across both near-native and non-native poses.

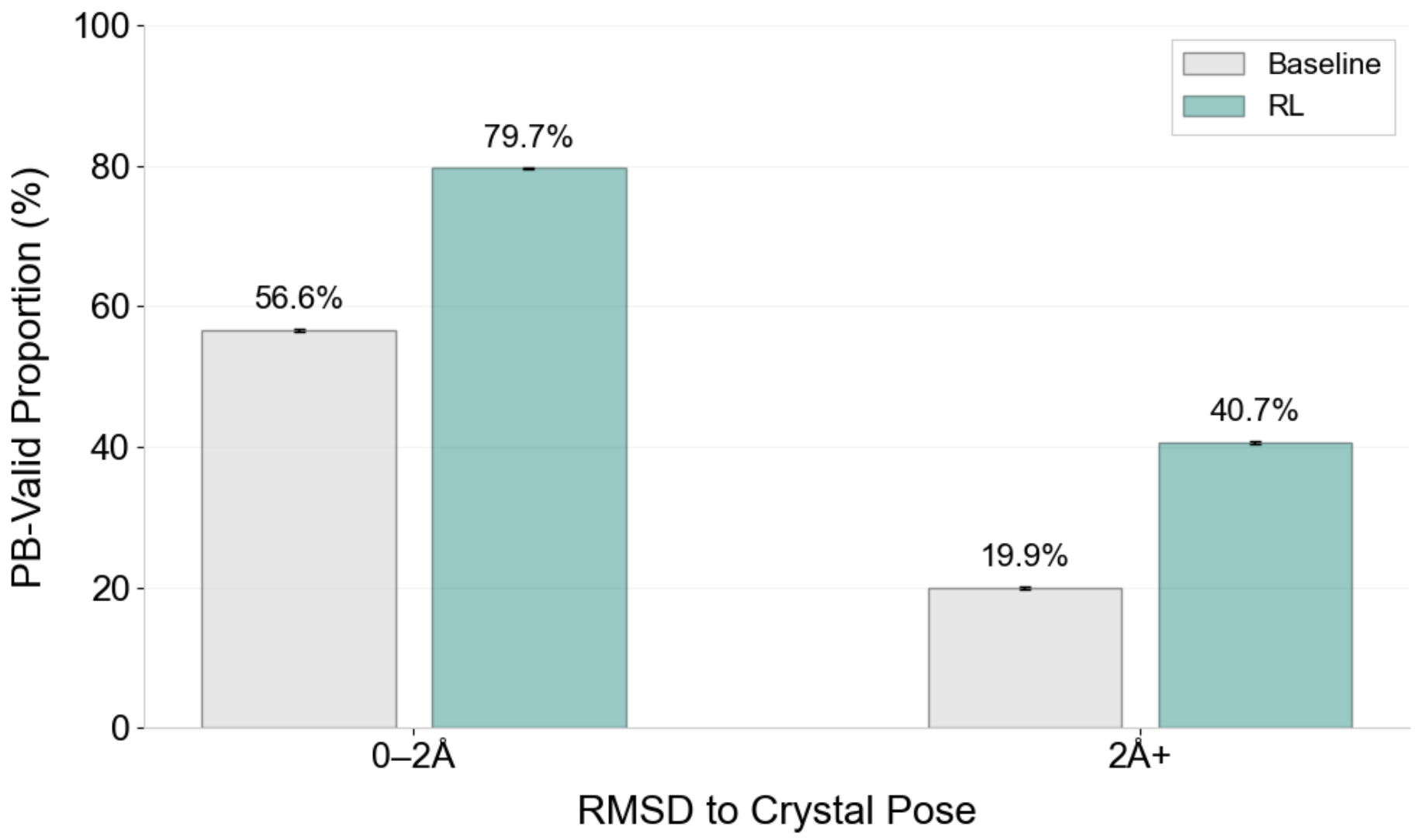

Fig. S8: Proportion of PB-valid poses stratified by RMSD to the crystal pose. Error bars show standard error across runs.

S6 Per-Complex PB-Valid Pose Comparison

Figure S9 shows the per-complex number of PB-valid poses for the overall set of poses generated for both the baseline and RL models. Each point corresponds to a single complex, with lines connecting paired baseline and RL results. Green lines indicate cases where RL generates more valid poses than the baseline, while red lines indicate cases where RL generates fewer. The violin plots show the distribution across complexes, and horizontal bars indicate the median.

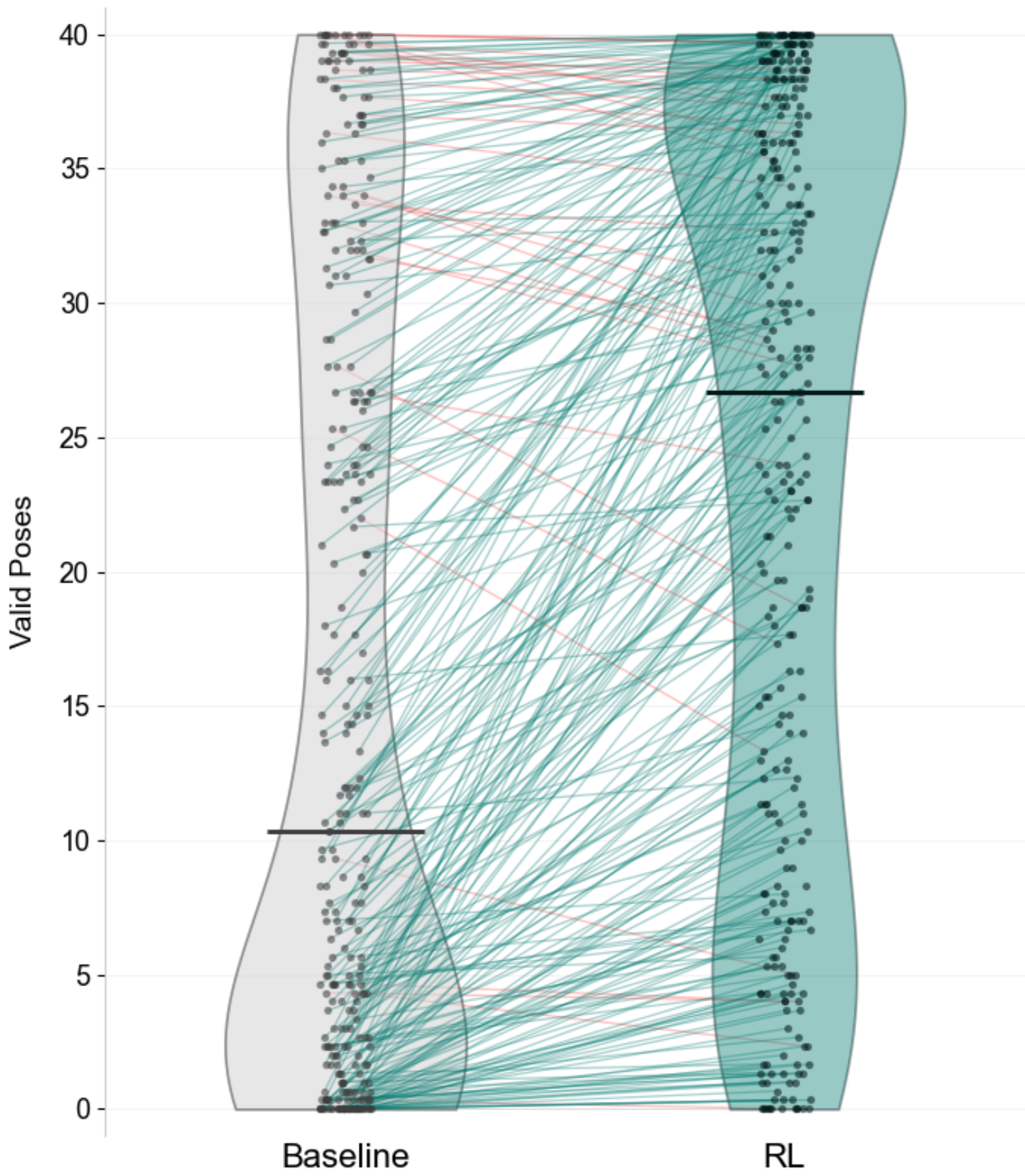

Fig. S9: Comparison of PB-valid poses between baseline and RL. Each point represents a complex, with lines connecting paired results. Green lines indicate improvements under RL, while red lines indicate decreases. Violins show the distribution across complexes, and horizontal bars indicate medians.

S7 Stratified Results by Sequence Similarity to the Training Set

To evaluate generalization performance, we stratify the PoseBusters benchmark set by sequence similarity to the training set. Results show that RL improves performance over the baseline across all similarity bins, with the strongest gains observed for the lowest similarity targets (0–30%).

Table S1: Success rates under increasingly strict quality criteria across sequence similarity bins (Top-1).

	0–30% (n=108)		30–95% (n=77)		95–100% (n=123)	
**Metric**	**Baseline**	**RL**	**Baseline**	**RL**	**Baseline**	**RL**
RMSD ≤ 2 Å	56.8	**60.5** ^{ns}	70.1	**72.7** ^{ns}	73.4	**74.3** ^{ns}
& PB-valid	31.5	**46.6** ****	50.2	**61.5** **	56.6	**67.8** ****
& ≥50% IR	23.8	**37.7** ****	43.7	**52.8** *	49.1	**58.3** ***
& ≥75% IR	13.0	**23.8** ****	38.1	**39.0** ^{ns}	36.0	**44.4** ***

Significance assessed using McNemar's test comparing Baseline and RL success outcomes across complexes. ns: not significant ($p \geq 0.05$); $^{*}p < 0.05$; $^{**}p < 0.01$; $^{***}p < 0.001$; $^{****}p < 0.0001$.

Table S2: Success rates under increasingly strict quality criteria across sequence similarity bins (Oracle).

	0–30% (n=108)		30–95% (n=77)		95–100% (n=123)	
**Metric**	**Baseline**	**RL**	**Baseline**	**RL**	**Baseline**	**RL**
RMSD ≤ 2 Å	68.2	**76.2** ****	80.5	**87.0** **	85.1	**91.1** ***
& PB-valid	49.1	**67.0** ****	68.0	**82.7** ****	79.9	**89.4** ****
& ≥50% IR	43.8	**61.7** ****	64.1	**77.1** ****	75.1	**84.6** ****
& ≥75% IR	31.5	**50.6** ****	56.7	**65.4** **	64.2	**77.0** ****

Significance assessed using McNemar's test comparing Baseline and RL success outcomes across complexes. ns: not significant ($p \geq 0.05$); $^{*}p < 0.05$; $^{**}p < 0.01$; $^{***}p < 0.001$; $^{****}p < 0.0001$.

S7.1 Minimized Structures

We also stratify the PoseBusters benchmark set by sequence similarity for poses which have been minimized and re-ranked using Gnina[7].

Table S3: Success rates under increasingly strict quality criteria for minimized structures (Top-1).

	0–30% (n=108)		30–95% (n=77)		95–100% (n=123)	
**Metric**	**Baseline**	**RL**	**Baseline**	**RL**	**Baseline**	**RL**
RMSD ≤ 2 Å	**70.7** ^{ns}	69.4	78.4	**84.0** *	82.7	**87.3** **
& PB-valid	**68.2** ^{ns}	66.7	76.6	**81.8** ^{ns}	81.3	**86.2** **
& ≥50% IR	**61.4** *	54.6	69.7	**75.8** ^{ns}	69.1	**77.5** ***
& ≥75% IR	**48.1** ^{ns}	45.4	53.7	**61.5** **	56.4	**61.2** ^{ns}

Significance assessed using McNemar's test comparing Baseline and RL success outcomes across complexes. ns: not significant ($p \geq 0.05$); $^{*}p < 0.05$; $^{**}p < 0.01$; $^{***}p < 0.001$; $^{****}p < 0.0001$.

Table S4: Success rates under increasingly strict quality criteria for minimized structures (Oracle).

	0–30% (n=108)		30–95% (n=77)		95–100% (n=123)	
**Metric**	**Baseline**	**RL**	**Baseline**	**RL**	**Baseline**	**RL**
RMSD $\leq$ 2 Å	76.9	**81.5** *	86.6	**91.3** *	88.1	**93.0** **
& PB-valid	75.3	**79.6** *	85.3	**90.5** *	88.1	**93.0** **
& $\geq$50% IR	73.1	**73.5** ^{ns}	81.4	**86.1** ^{ns}	82.4	**88.1** **
& $\geq$75% IR	63.6	**67.3** ^{ns}	73.6	**73.6** ^{ns}	71.3	**79.7** ***

Significance assessed using McNemar's test comparing Baseline and RL success outcomes across complexes. ns: not significant ($p \geq 0.05$); $^{*}p < 0.05$; $^{**}p < 0.01$; $^{***}p < 0.001$; $^{****}p < 0.0001$.

S8 Stratified Results by PDBbind SuCOS-Pocket Similarity

We stratify performance by the maximum ligand-pocket similarity between each PoseBusters complex and the PDBbind training set. Success is defined as RMSD $\leq$ 2 Å and PB-valid for the top-1 ranked pose. RL improves performance across most ligand-pocket similarity bins, with the most notable improvements occurring for complexes with lower similarity to the training set.

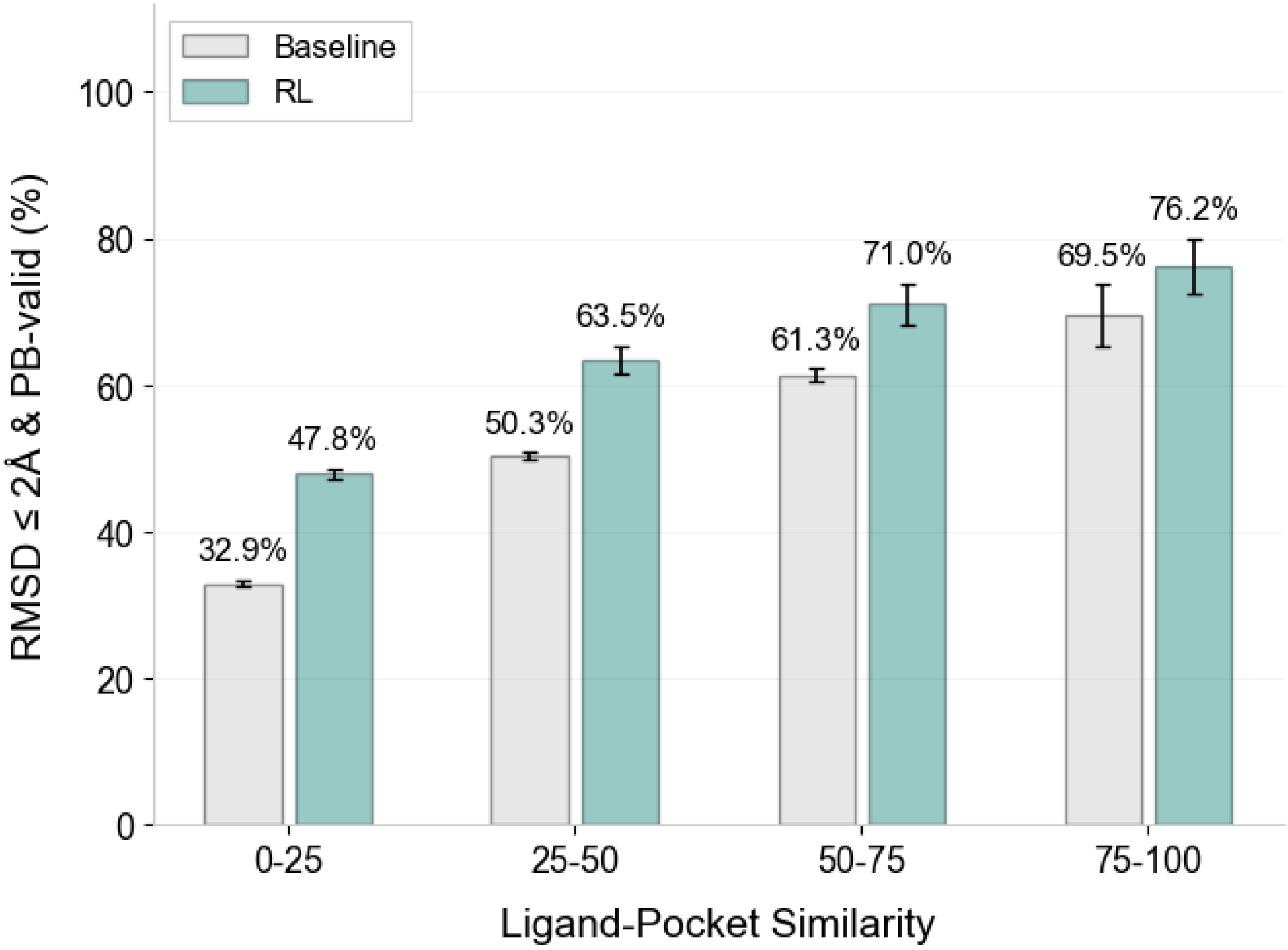

Fig. S10: Top-1 success rates stratified by ligand-pocket similarity to PDBbind. Success is defined as RMSD $\leq$ 2 Å and PB-valid. Complexes are grouped by maximum ligand-pocket similarity to the PDBbind training set. Error bars show standard error across independent runs.

S9 Results for Docking into Apo Structures

To evaluate performance in a more realistic docking setting, we assess both DiffDock-Pocket and DiffDock-Pocket RL using predicted apo receptor structures from the AlphaFold Database. Unlike docking into experimentally determined holo structures, this removes access to the ligand-bound receptor conformation and introduces additional uncertainty from receptor modelling error and binding-site rearrangement.

This analysis is restricted to the 284 PoseBusters benchmark targets for which a corresponding predicted structure was available. For each target, we dock the corresponding ligand into the predicted apo structure and evaluate performance against the experimentally determined ligand pose from the holo complex.

Overall, RL fine-tuning improves performance across almost all apo-structure evaluation criteria, with the clearest gains observed for PB-valid poses and poses that also recover native interactions (Table S5). This trend is also observed after minimization (Table S6). Although the baseline achieves higher Top-1 RMSD-only performance in the unminimized apo setting, RL performs better once physical validity and interaction recovery are included, particularly under the Oracle evaluation (Tables S5 and S6).

Table S5: Success rates under increasingly strict quality criteria for apo structures (n=284). RL improves performance for physically valid and interaction-recovery criteria, while the baseline achieves higher Top-1 RMSD-only performance.

	**Top-1 (%)**		**Oracle (%)**	
**Metric**	**Baseline**	**RL**	**Baseline**	**RL**
RMSD ≤ 2Å	**43.0 ± 0.6** ****	37.0 ± 2.1	60.9 ± 0.6	**64.4 ± 0.7** **
& PB-valid	15.5 ± 0.9	**23.2 ± 0.9** ****	35.0 ± 1.4	**46.4 ± 2.0** ****
& ≥50% IR	12.3 ± 1.4	**17.5 ± 0.2** ****	29.2 ± 1.3	**37.6 ± 1.2** ****
& ≥75% IR	9.5 ± 0.4	**13.6 ± 0.5** ***	23.8 ± 2.2	**31.1 ± 0.2** ****

Values are reported as mean ± standard deviation across runs. Significance assessed using McNemar's test comparing Baseline and RL success outcomes across complexes. ns: not significant ($p \geq 0.05$); $^{*}p < 0.05$; $^{**}p < 0.01$; $^{***}p < 0.001$; $^{****}p < 0.0001$.

Table S6: Success rates under increasingly strict quality criteria for minimized apo structures (n=284). RL improves Oracle performance across all criteria after minimization, while Top-1 improvements are smaller and not statistically significant.

	**Top-1 (%)**		**Oracle (%)**	
**Metric**	**Baseline**	**RL**	**Baseline**	**RL**
RMSD ≤ 2Å	42.8 ± 1.1	**43.2 ± 2.3** ^{ns}	56.7 ± 1.1	**61.3 ± 1.6** ****
& PB-valid	36.6 ± 1.1	**38.3 ± 2.1** ^{ns}	50.7 ± 0.9	**55.5 ± 1.0** ****
& ≥50% IR	25.0 ± 0.9	**27.2 ± 2.4** ^{ns}	41.3 ± 0.5	**46.7 ± 0.9** ****
& ≥75% IR	18.5 ± 0.7	**20.3 ± 0.9** ^{ns}	30.4 ± 0.9	**34.7 ± 0.4** ***

Values are reported as mean ± standard deviation across runs. Significance assessed using McNemar's test comparing Baseline and RL success outcomes across complexes. ns: not significant ($p \geq 0.05$); $^{*}p < 0.05$; $^{**}p < 0.01$; $^{***}p < 0.001$; $^{****}p < 0.0001$.

S9.1 Stratified Apo Results by Sequence Similarity to the Training Set

To evaluate generalization performance on apo structures, we stratify the PoseBusters benchmark set by sequence similarity to the training set.

Table S7: Success rates under increasingly strict quality criteria across sequence similarity bins for apo structures (Top-1).

	0–30% (n=100)		30–95% (n=68)		95–100% (n=116)	
**Metric**	**Baseline**	**RL**	**Baseline**	**RL**	**Baseline**	**RL**
RMSD ≤ 2 Å	**36.7** *	31.0	**42.2** *	35.3	**48.9** **	43.1
& PB-valid	11.3	**18.3** **	14.2	**22.5** **	19.8	**27.9** ****
& $\geq$50% IR	8.7	**14.3** *	13.2	**17.6** ^{ns}	14.9	**20.1** **
& $\geq$75% IR	6.0	**10.3** *	11.3	**15.7** ^{ns}	11.5	**15.2** *

Significance assessed using McNemar's test comparing Baseline and RL success outcomes across complexes. ns: not significant ($p \geq 0.05$); $^{*}p < 0.05$; $^{**}p < 0.01$; $^{***}p < 0.001$; $^{****}p < 0.0001$.

Table S8: Success rates under increasingly strict quality criteria across sequence similarity bins for apo structures (Oracle).

	0–30% (n=100)		30–95% (n=68)		95–100% (n=116)	
**Metric**	**Baseline**	**RL**	**Baseline**	**RL**	**Baseline**	**RL**
RMSD ≤ 2 Å	54.7	**60.3** **	**58.3** ^{ns}	57.8	67.8	**71.8** *
& PB-valid	29.3	**43.0** ****	35.8	**40.7** ^{ns}	39.4	**52.6** ****
& $\geq$50% IR	23.3	**33.3** ****	31.4	**35.3** ^{ns}	33.0	**42.5** ****
& $\geq$75% IR	19.0	**26.0** ***	25.5	**29.4** ^{ns}	27.0	**36.5** ****

Significance assessed using McNemar's test comparing Baseline and RL success outcomes across complexes. ns: not significant ($p \geq 0.05$); $^{*}p < 0.05$; $^{**}p < 0.01$; $^{***}p < 0.001$; $^{****}p < 0.0001$.

S9.2 Minimized Apo Structures

To evaluate the impact of minimization on apo generalization performance, we also stratify the PoseBusters benchmark set by sequence similarity.

Table S9: Success rates under increasingly strict quality criteria for minimized apo structures (Top-1).

	0–30% (n=100)		30–95% (n=68)		95–100% (n=116)	
**Metric**	**Baseline**	**RL**	**Baseline**	**RL**	**Baseline**	**RL**
RMSD $\leq$ 2 Å	41.3	**42.3** ^{ns}	41.7	**42.6** ^{ns}	**44.8** ^{ns}	44.3
& PB-valid	33.3	**36.7** ^{ns}	38.2	**39.7** ^{ns}	38.5	**38.8** ^{ns}
& $\geq$50% IR	24.3	**27.7** ^{ns}	24.5	**27.9** ^{ns}	25.9	**26.4** ^{ns}
& $\geq$75% IR	18.3	**19.3** ^{ns}	20.1	**22.1** ^{ns}	17.8	**20.1** ^{ns}

Significance assessed using McNemar's test comparing Baseline and RL success outcomes across complexes. ns: not significant ($p \geq 0.05$); $^{*}p < 0.05$; $^{**}p < 0.01$; $^{***}p < 0.001$; $^{****}p < 0.0001$.

Table S10: Success rates under increasingly strict quality criteria for minimized apo structures (Oracle).

	0–30% (n=100)		30–95% (n=68)		95–100% (n=116)	
**Metric**	**Baseline**	**RL**	**Baseline**	**RL**	**Baseline**	**RL**
RMSD $\leq$ 2 Å	56.0	**61.3** **	53.9	**54.9** ^{ns}	58.9	**64.9** **
& PB-valid	51.7	**55.7** ^{ns}	47.5	**50.5** ^{ns}	51.7	**58.3** ***
& $\geq$50% IR	45.0	**49.7** ^{ns}	39.7	**43.6** *	39.1	**46.0** **
& $\geq$75% IR	31.3	**35.7** ^{ns}	32.4	**33.8** ^{ns}	28.4	**34.5** **

Significance assessed using McNemar's test comparing Baseline and RL success outcomes across complexes. ns: not significant ($p \geq 0.05$); $^{*}p < 0.05$; $^{**}p < 0.01$; $^{***}p < 0.001$; $^{****}p < 0.0001$.

S10 Confidence Score and Pose Quality

DiffDock-Pocket uses a confidence model to rank generated poses. Since the confidence model was not retrained during RL fine-tuning, we analyse whether this fixed confidence score remains correlated with pose quality for DiffDock-Pocket RL.

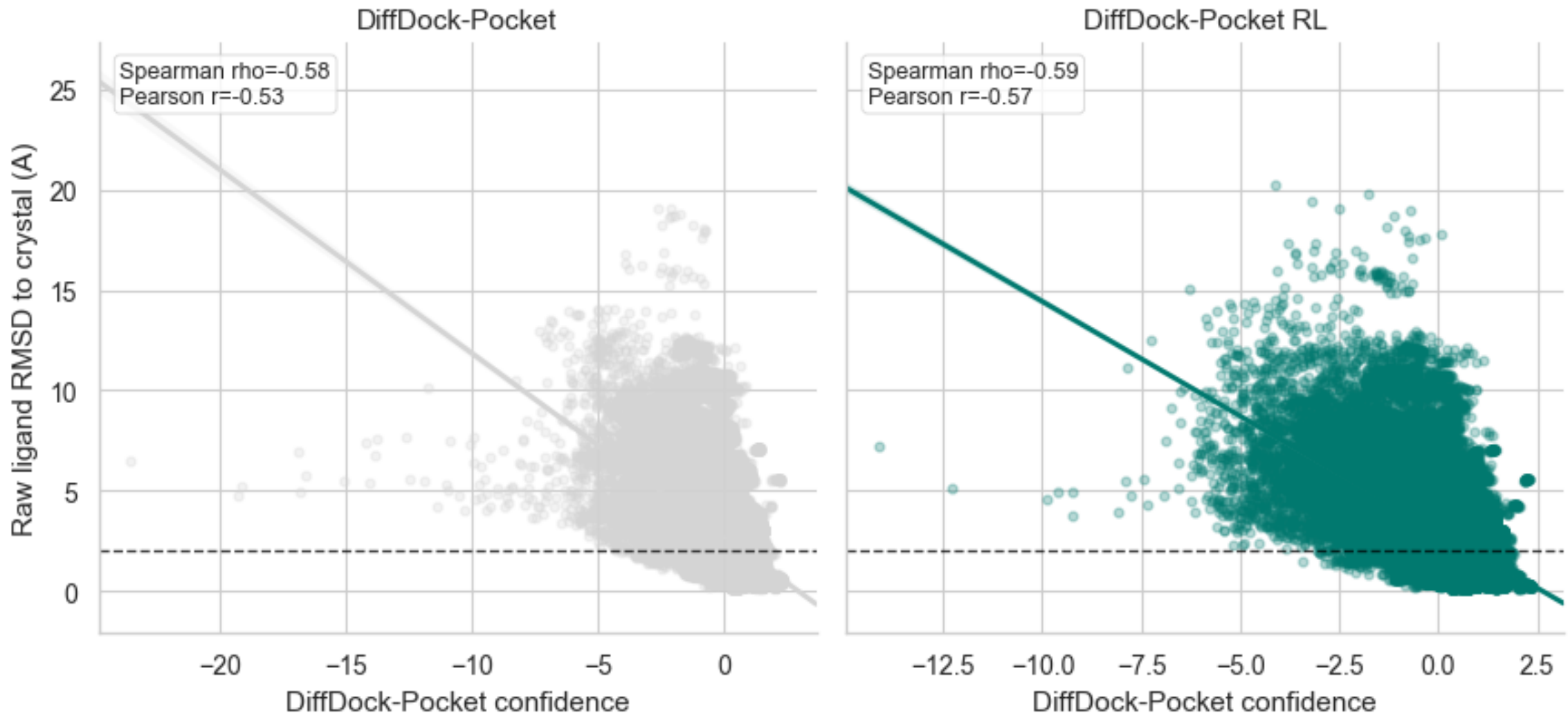

Fig. S11: Confidence score versus RMSD for all generated poses. Confidence remains correlated with pose quality for both DiffDock-Pocket and DiffDock-Pocket RL. For DiffDock-Pocket RL, the Spearman correlation between confidence and RMSD is −0.59, compared with −0.58 for the original DiffDock-Pocket model. Since lower RMSD indicates better pose accuracy, this shows that higher-confidence poses generally have lower RMSD.

Across all generated poses, the confidence score remains informative after RL fine-tuning (Fig. S11). The correlation between confidence and RMSD is similar for DiffDock-Pocket and DiffDock-Pocket RL, indicating that the existing confidence model still captures information about pose accuracy despite the shift in the generated pose distribution.

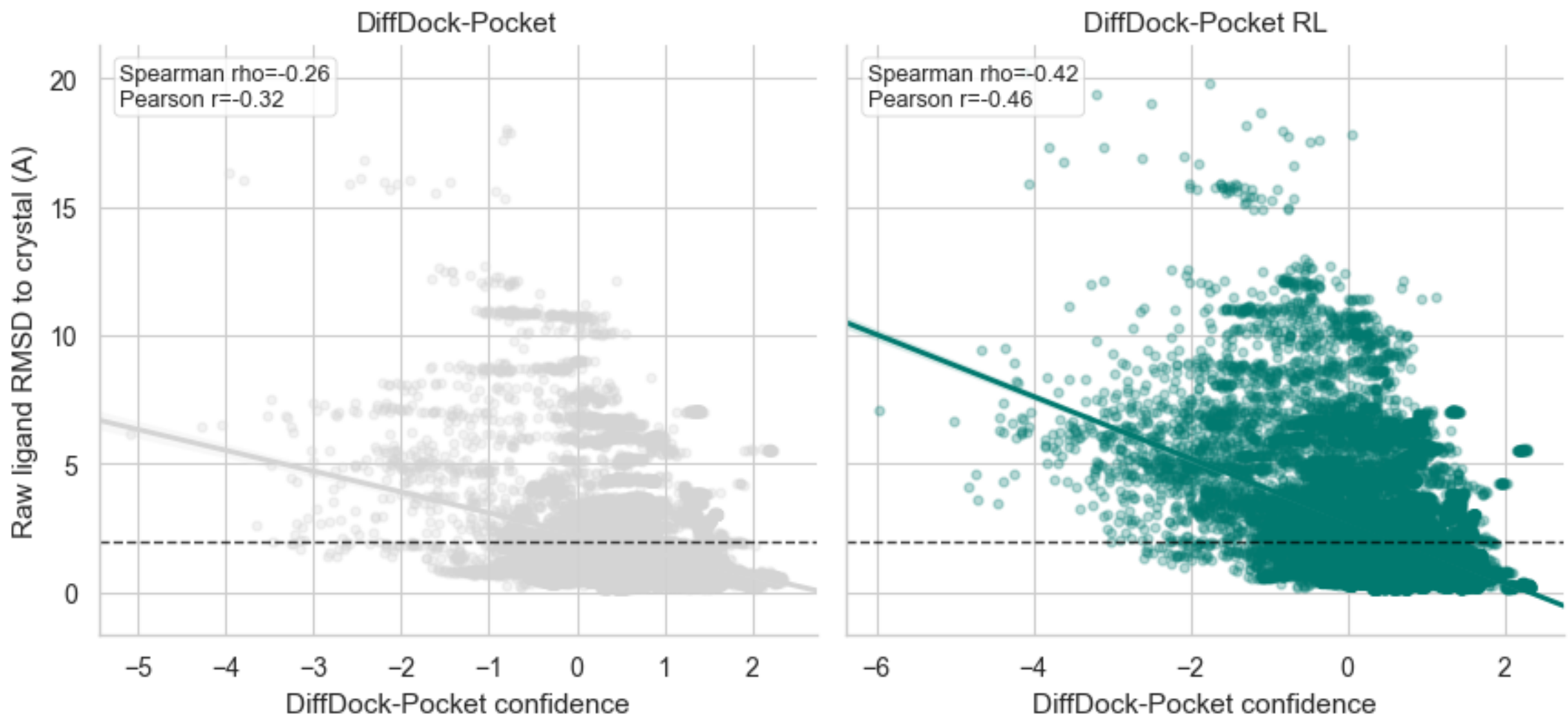

Fig. S12: Confidence score versus RMSD for PB-valid poses only. When considering only PB-valid poses, confidence remains correlated with RMSD. The correlation is stronger for DiffDock-Pocket RL than for the baseline, with Spearman correlation changing from −0.26 for DiffDock-Pocket to −0.42 for DiffDock-Pocket RL.

We also repeat the analysis using only PB-valid poses (Fig. S12). This removes physically invalid

poses and tests whether the confidence score still distinguishes better and worse poses within the physically plausible subset. Confidence remains correlated with RMSD in this setting, suggesting that it carries information about pose accuracy beyond simply identifying invalid structures.

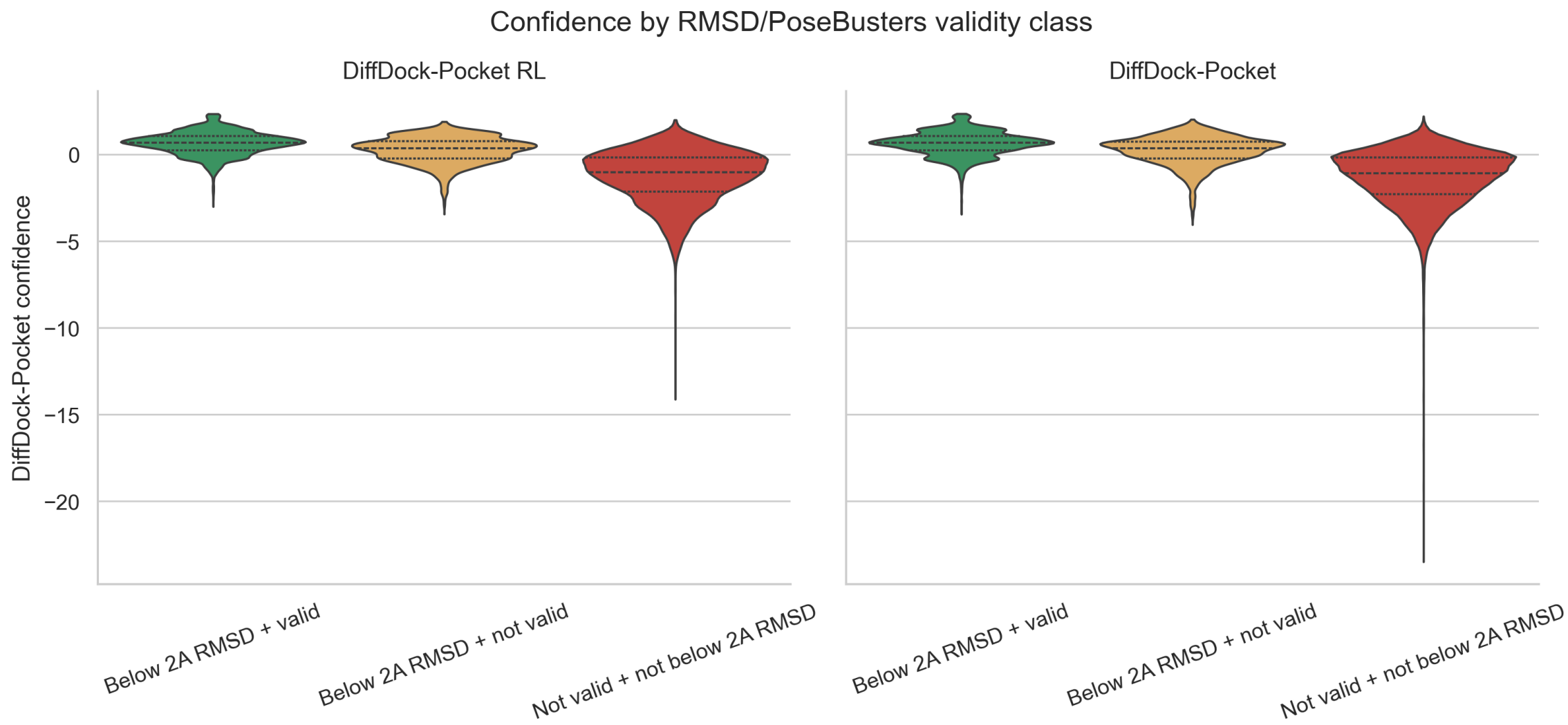

Fig. S13: Confidence score by RMSD and PoseBusters validity class. Poses that are both PB-valid and below 2 Å RMSD receive the highest confidence scores. Poses below 2 Å RMSD but not PB-valid receive slightly lower scores, while poses that are neither PB-valid nor below 2 Å RMSD receive the lowest scores.

Finally, we compare confidence scores across pose-quality classes (Fig. S13). For both DiffDock-Pocket and DiffDock-Pocket RL, the confidence model assigns the highest scores to poses that are both accurate and physically valid, and the lowest scores to poses that are neither accurate nor physically valid.

Overall, these results show that the DiffDock-Pocket confidence score remains correlated with pose quality after RL fine-tuning (Figs. S11–S13). However, the confidence model does not always identify the best generated pose, as shown by the gap between Oracle and Top-1 success rates throughout the manuscript. This suggests that RL improves the ability of the model to generate high-quality poses, while pose ranking remains a separate limitation.

S11 Overlap in Success Sets Across Sequence Identity Groups

Figures S14 to S16 show how the success sets of the original DiffDock-Pocket and the RL-trained DiffDock-Pocket model overlap across three sequence identity groups. As the success criteria becomes more strict, the success sets shrink and the overlap typically decreases. This creates greater divergence between the models, meaning one model succeeds on complexes where the other fails. Across all groups, the divergence increasingly favours RL under strict criteria. This effect is strongest for low sequence identity targets, which are the most out of distribution.

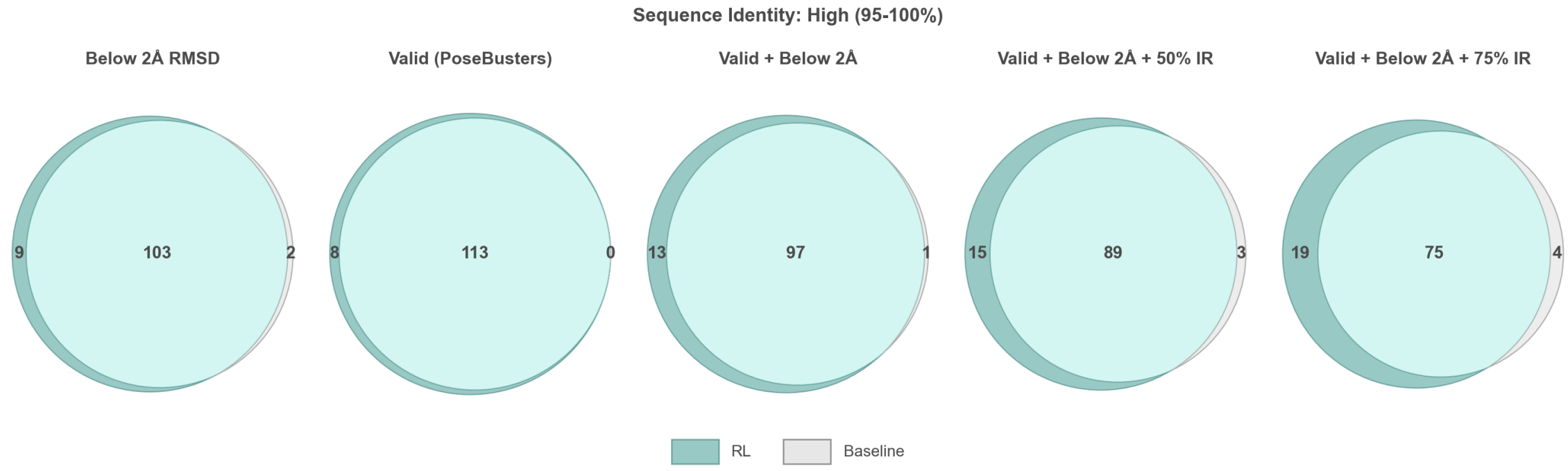

Fig. S14: Overlap in success sets between the baseline DiffDock-Pocket model and the RL trained model for high sequence identity targets in the range 95%–100%. $N = 123$.

For sequence identity in the range 95%–100%, many successes are shared between the two models across all criteria. This pattern is consistent with an in-distribution regime where the baseline already performs well. As the success criteria become more strict, the sets shrink but most successes remain shared. RL still contributes additional successes, and these appear most clearly when success requires PB-validity and RMSD ≤2Å as well as recovery of 75% of ground-truth interactions.

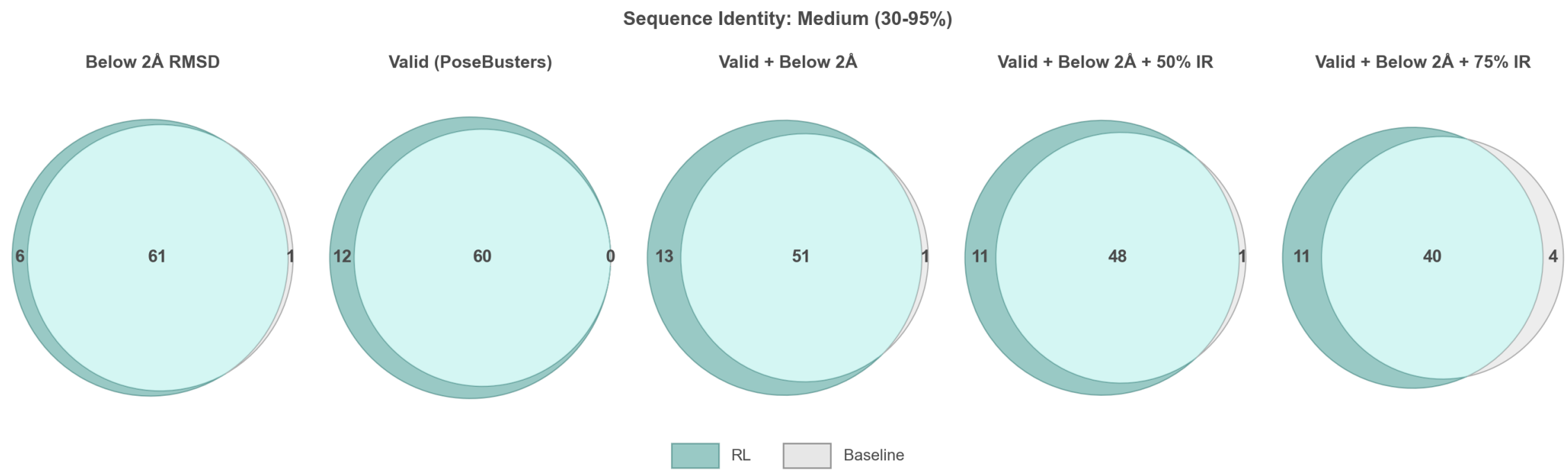

Fig. S15: Overlap in success sets between the baseline DiffDock-Pocket model and the RL trained model for medium sequence identity targets in the range 30%–95%. $N = 77$.

For sequence identity in the range 30%–95%, when success is defined by PB validity and RMSD below 2 Å, the overlap decreases and model specific successes become more frequent. This indicates that the harder requirement is not simply generating a near native pose or generating a plausible pose in isolation, but generating a pose that satisfies both at the same time. RL improves this joint success more consistently than the baseline, and the gap becomes more visible once interaction recovery thresholds are included.

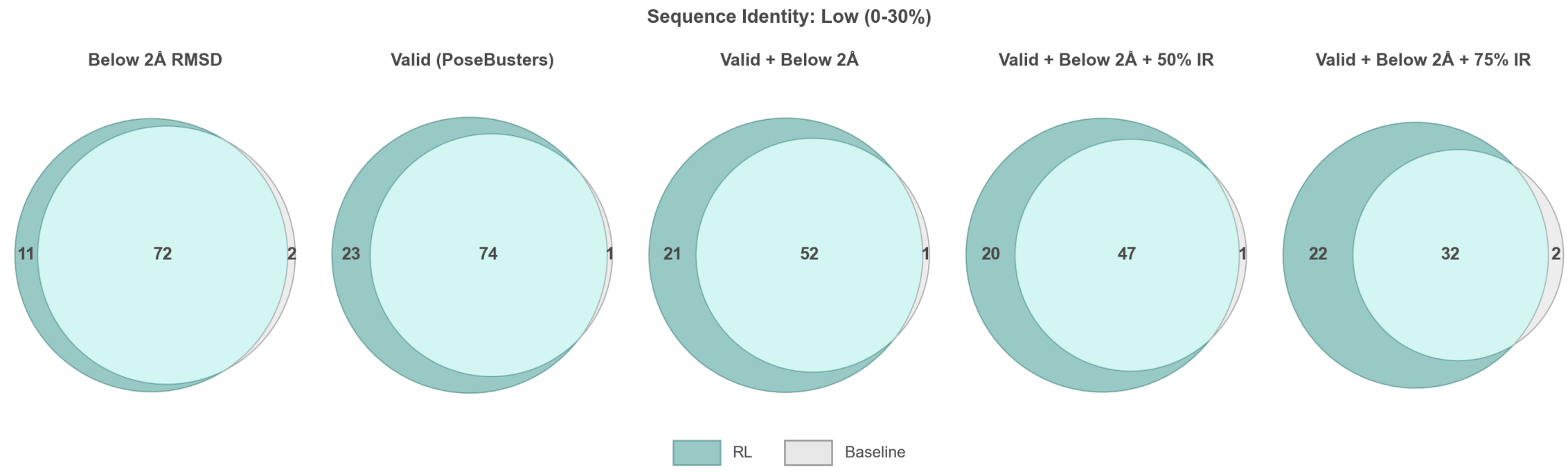

Fig. S16: Overlap in success sets between the baseline DiffDock-Pocket model and the RL trained model for low sequence identity targets in the range 0%–30%. $N = 108$.

For sequence identity in the range 0%–30%, the success sets diverge substantially as the criteria become more strict. The baseline retains relatively few successes once PB validity and RMSD below 2 Åare required simultaneously, and it contracts further when interaction recovery thresholds are added. In contrast, RL preserves a much larger successful set under these strict criteria, producing many RL only successes. This widening divergence supports the conclusion that reinforcement learning most strongly improves performance on the most out of distribution targets, particularly when success requires joint near nativeness, physical plausibility, and interaction recovery.

S12 Comparison to AlphaFold3

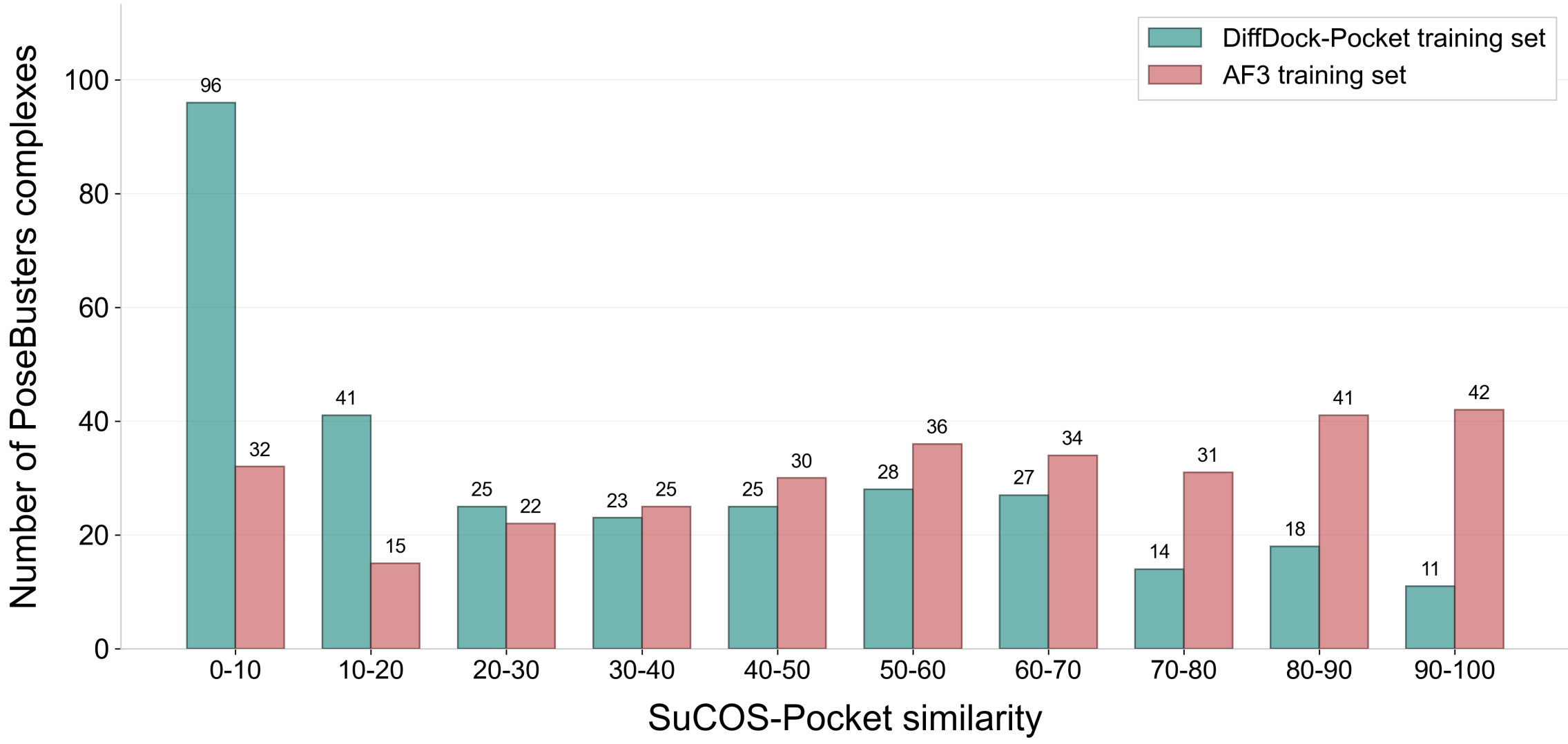

Fig. S17: Similarity of PoseBusters complexes to the DiffDock-Pocket and AlphaFold3 training sets. PoseBusters complexes are stratified by their maximum SuCOS-pocket similarity to complexes in each training set. PoseBusters has a substantially higher proportion of similar complexes with the AlphaFold3 training set than with the DiffDock-Pocket training set.

Direct comparison between DiffDock-Pocket RL++ and AlphaFold3 is complicated by differences in overlap between their respective training sets and PoseBusters. As shown in Fig. S17, PoseBusters has substantially greater overlap with the AlphaFold3 training set than with the DiffDock-Pocket training set. In particular, many more PoseBusters complexes fall into the highest ligand-pocket similarity bins for AlphaFold3, while many more fall into the lowest similarity bins for DiffDock-Pocket. This makes a direct comparison with AlphaFold3 informative but not strictly like-for-like. Nevertheless, DiffDock-Pocket RL++ performs comparably to AlphaFold3, achieving 78.2% RMSD $\leq$ 2Å and PB-valid poses, compared with 73.1% for AlphaFold3 without ground-truth structural information and 84.4% for AlphaFold3 when the pocket is specified.

S13 Training Hyperparameters

Table S11: Training hyperparameters for reinforcement learning fine-tuning of DiffDock-Pocket.

**Hyperparameter**	**Value**
**RL optimisation**	
Expert loss weight λ	0.03
Learning rate	1×10^{-4}
Optimizer	Adam
PPO clip range ϵ	0.3
Gradient clipping (norm)	2.0
Discount factor γ	0.95
**Diffusion / trajectory**	
Reverse diffusion steps	20
Expert-step cutoff T_E	9
Branching window	$t \in \{8, \ldots, 5\}$
Branching factor	2
**Sampling**	
Samples per complex	4
Complexes per batch	12
Policy updates per batch	3

S14 Comparison with Vina-Generated and Experimentally Observed Poses

To compare the energetic plausibility of generated poses, we compute the mean Vina energy for experimentally observed ligand poses, poses generated directly by Vina, and poses generated by DiffDock-Pocket before and after RL fine-tuning for the top-1 ranked poses. RL fine-tuning improves the mean Vina energy from -4.41 kcal/mol for the baseline to -6.77 kcal/mol. After minimization and re-ranking by the lowest vina energy pose (mirroring the way in which vina choose poses), DiffDock-Pocket RL achieves a mean Vina energy of -9.36 kcal/mol, close to the Vina-generated poses (-9.20 kcal/mol) and experimentally observed poses (-8.74 kcal/mol).

Table S12: Mean Vina energies for experimentally observed poses, Vina poses, DiffDock-Pocket baseline poses, and DiffDock-Pocket RL poses before and after minimization.

Method	Mean Vina energy
GT	−8.74
Vina	−9.20
DiffDock-Pocket baseline	−4.41
DiffDock-Pocket RL	−6.77
DiffDock-Pocket baseline minimized	−9.27
DiffDock-Pocket RL minimized	−9.36

We also compare the full distribution of Vina energies for poses generated directly by Vina and minimized DiffDock-Pocket RL poses. The two distributions show substantial overlap, with mean energies of -9.20 kcal/mol for Vina and -9.36 kcal/mol for minimized DiffDock-Pocket RL. This indicates that, after minimization, DiffDock-Pocket RL poses occupy a similar Vina-energy regime to Vina-generated poses.

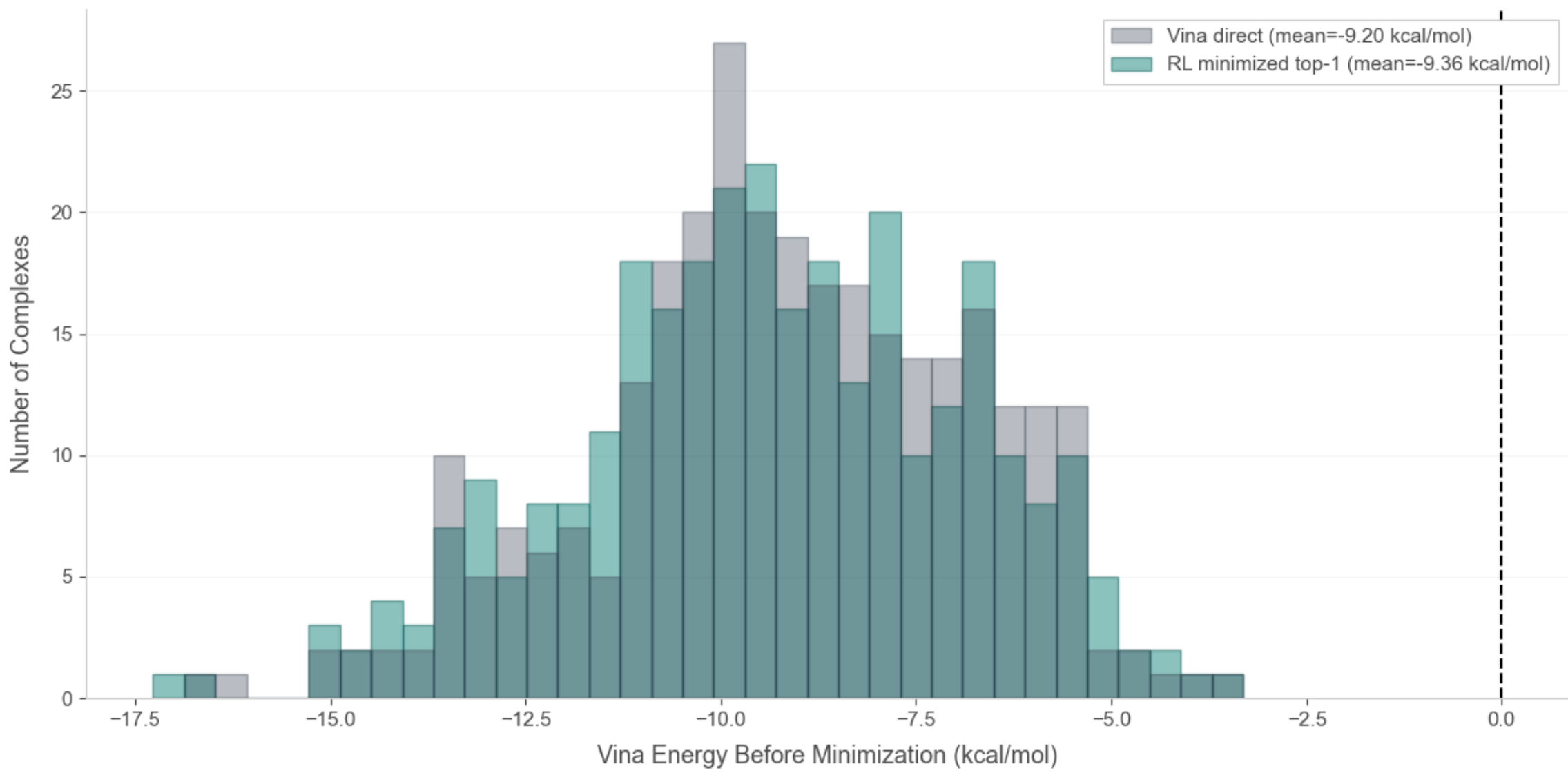

Fig. S18: Vina energy distributions for Vina-generated and minimized DiffDock-Pocket RL poses. Distributions are shown for poses generated directly by Vina and minimized DiffDock-Pocket RL top-1 poses, with mean energies of -9.20 kcal/mol and -9.36 kcal/mol, respectively.

References

[1] Oleg Trott and Arthur J. Olson. AutoDock Vina: Improving the speed and accuracy of docking with a new scoring function, efficient optimization, and multithreading. *Journal of Computational Chemistry*, 31(2):455–461, 2010. ISSN 1096-987X. doi: 10.1002/jcc.21334. URL https://onlinelibrary.wiley.com/doi/abs/10.1002/jcc.21334.

[2] Marcel L. Verdonk, Jason C. Cole, Michael J. Hartshorn, Christopher W. Murray, and Richard D. Taylor. Improved protein–ligand docking using GOLD. *Proteins: Structure, Function, and Bioinformatics*, 52(4):609–623, 2003. ISSN 1097-0134. doi: 10.1002/prot.10465. URL https://onlinelibrary.wiley.com/doi/abs/10.1002/prot.10465.

[3] Michael Plainer, Marcella Toth, Simon Dobers, Hannes Stark, Gabriele Corso, Céline Marquet, and Regina Barzilay. DiffDock-Pocket: Diffusion for pocket-level docking with sidechain flexibility. In *NeurIPS 2023 Workshop on New Frontiers of AI for Drug Discovery and Development*, 2023. URL https://openreview.net/forum?id=5Wt7qNRc5u.

[4] Alvaro Prat, Leo Zhang, Charlotte Deane, Yee Whye Teh, and Garrett M. Morris. SigmaDock: Untwisting molecular docking with fragment-based SE(3) diffusion. In *International Conference on Learning Representations*, 2026. URL https://openreview.net/forum?id=Vgm77U4ojX.

[5] Gengmo Zhou, Zhifeng Gao, Qiankun Ding, Hang Zheng, Hongteng Xu, Zhewei Wei, Linfeng Zhang, and Guolin Ke. Uni-Mol: A universal 3d molecular representation learning framework. In *International Conference on Learning Representations*, 2023. URL https://openreview.net/forum?id=6K2RM6wVqKu.

[6] Zhirui Liao, Ronghui You, Xiaodi Huang, Xiaojun Yao, Tao Huang, and Shanfeng Zhu. DeepDock: Enhancing ligand-protein interaction prediction by a combination of ligand and structure information. In *2019 IEEE International Conference on Bioinformatics and Biomedicine*, pages 311–317, San Diego, CA, USA, 2019. IEEE. doi: 10.1109/BIBM47256.2019.8983365. URL https://ieeexplore.ieee.org/document/8983365/.

[7] Andrew T. McNutt, Paul Francoeur, Rishal Aggarwal, Tomohide Masuda, Rocco Meli, Matthew Ragoza, Jocelyn Sunseri, and David Ryan Koes. GNINA 1.0: molecular docking with deep learning. *Journal of Cheminformatics*, 13(1):43, June 2021. ISSN 1758-2946. doi: 10.1186/s13321-021-00522-2. URL https://doi.org/10.1186/s13321-021-00522-2.
